# Genetic Diversity, Predictive Protein Structures, and Interaction Networks of Cysteine-Rich Receptor-Like Kinases in *Arabidopsis thaliana*

**DOI:** 10.34133/csbj.0043

**Published:** 2026-04-08

**Authors:** Jente Stouthamer, Danilo Pereira, Sergio Martin-Ramirez, Sumanth Mutte, G. Adam Mott, Elwira Smakowska-Luzan

**Affiliations:** ^1^ Laboratory of Biochemistry, Wageningen University and Research, 6708 WE Wageningen, The Netherlands.; ^2^ Department of Biological Sciences, University of Toronto Scarborough, Toronto, Canada.; ^3^ Department of Cell and Systems Biology, University of Toronto, Toronto, Canada.

## Abstract

Cysteine-rich receptor-like kinases (CRKs) are a large subfamily of plant receptor-like kinases implicated in immunity and development, yet their ligands, interaction partners, and mechanistic roles remain poorly defined. We combined population-genetic analyses and AlphaFold-based structural prediction to characterize the *Arabidopsis thaliana* CRK family. Phylogenetic reconstruction from 69 natural accessions resolved 5 well-supported CRK clades. Nucleotide diversity (π) and neutrality tests revealed heterogeneous diversity across loci, with evidence of both positive and negative selection pressure acting on different CRKs. AlphaFold models of CRK extracellular domains (ECDs) recapitulate the DUF26 structure observed in plasmodesmata localizing protein (PDLP)5/PDLP8 and ginkbilobin-2 but display distinct biochemical properties and disulfide-bond topologies. Pairwise AlphaFold dimer modeling of all 780 CRK–ECD combinations produced 145 high-confidence interaction models; ~78% of these adopt a shared dimer conformation characterized by an extended intermolecular β-sheet at the interface. Integrating evolutionary and structural approaches reveal clade-specific selective regimes and conserved structural features of CRK–ECDs that likely underpin receptor–receptor interactions. Predicted high-confidence dimer interfaces suggest a general mode of CRK–ECD association that can guide targeted biochemical and genetic validation, accelerating functional dissection of this important receptor family.

## Introduction

Plant cells continuously perceive and respond to fluctuating environmental cues via extensive receptor-mediated signaling networks. *Arabidopsis thaliana* encodes >600 receptor-like kinases (RLKs), among which the cysteine-rich receptor-like kinases (CRKs; 44 members in Arabidopsis) constitute a major RLK subfamily implicated in immunity, stress response, and development [1]. Like canonical receptors, CRKs are also modular membrane proteins. At the N-terminus, CRKs have a signal peptide that directs the protein to the plasma membrane. Following that is the extracellular domain (ECD) containing 2 tandem domains of unknown function (DUF26-A and DUF26-B) defined by a conserved cysteine motif (C-8X-C-2X-C) [2]. Canonically, as demonstrated for other RLKs, the ECDs are most likely required for the perception of signaling molecules and complex formation with other RLKs [3]. Due to the presence of cysteines in the ECDs, members of the CRK family have long been hypothesized as sensors for reactive oxygen species (ROS) in plants. A long juxtamembrane region (average ~35 AA) rich in proline residues connects the ECD to the single transmembrane domain (TMD) composed of an α-helixes. Intracellularly, another juxtamembrane region rich in lysine and arginine residues connects the TMD to the serine/threonine kinase domain (KD). In general, both the juxtamembrane regions and the TMD can regulate protein activity, localization, and interaction at the plasma membrane. The conserved KD is required for intracellular interactions and signal propagation through autophosphorylation and the transphosphorylation of target proteins [4,5]. The KD is highly conserved among CRKs, including regions important for function and regulation: the adenosine triphosphate-binding site, catalytic loop, and activation loop.

Functional specificity among RLKs is generally conferred by ECD architecture, which mediates ligand recognition and receptor–receptor interactions [4,6,7]. The extracellular DUF26 domains are also found in plasmodesmata-localized proteins (PDLPs) and cysteine-rich repeat secreted proteins (CRRSPs) [1,8,9]. Consistent with activation mechanisms established for other RKs, CRKs are proposed to oligomerize and engage extracellular ligands to trigger intracellular signaling. However, to date, there are very few studies demonstrating interactions among different members of the CRK family. For example, CRK28 forms homo- and hetero-oligomers with CRK29, yet the mechanistic consequences of these interactions for specific immune or developmental outputs remain unresolved [10]. Overall, CRK ligands and interaction partners are unknown, limiting functional insight into this family.

Computational biochemical and structural prediction provides a tractable route to infer RLK architecture, potential interaction interfaces, and dimerization modes, thereby guiding hypothesis generation and experimental design [11,12]. Advances in deep-learning structure prediction, exemplified by AlphaFold (AF), now permit high-accuracy modeling of individual proteins and of protein–protein assemblies, offering atomic-level hypotheses for binding interfaces and structural compatibility [13]. Although predictive models do not replace biochemical and genetic validation, they enable prioritization of experiments and interpretation of sequence–structure–function relationships.

Here, we combine population-genetics and structure-prediction approaches to characterize the Arabidopsis CRK family. Using a panel of 69 natural accessions, we reconstructed the CRK phylogeny and confirmed 5 well-supported phylogenetic clades. Within each clade, we quantified nucleotide diversity (π) and performed selection neutrality tests, revealing heterogeneous diversity profiles across CRKs and signatures of both purifying and balancing selection acting at different loci. To explore potential functional determinants, we analyzed sequence features and AF-predicted structures of CRK–ECDs. Predicted CRK–ECD folds are broadly similar to experimentally determined DUF26-containing structures (PDLP5, PDLP8, and ginkbilobin-2) but show distinct glycosylation patterns and disulfide-bond topologies. We further generated pairwise CRK–ECD dimer models (780 combinations); AF Multimer produced 145 high-confidence dimer predictions, of which ~78% adopted a conserved dimer conformation. Many predicted dimers form an extended intermolecular β-sheet at the interface, suggesting a putative general interaction mechanism. These results define structural and evolutionary features of CRK–ECDs that prioritize candidates and interfaces for biochemical and genetic follow-up to elucidate CRK-mediated signaling. Moreover, this work offers a range of computational approaches to explore biochemical and structural features of the protein at the family level.

## Results

### CRK nucleotide sequence diversity, dynamics, and phylogeny

**Fig. 1.**
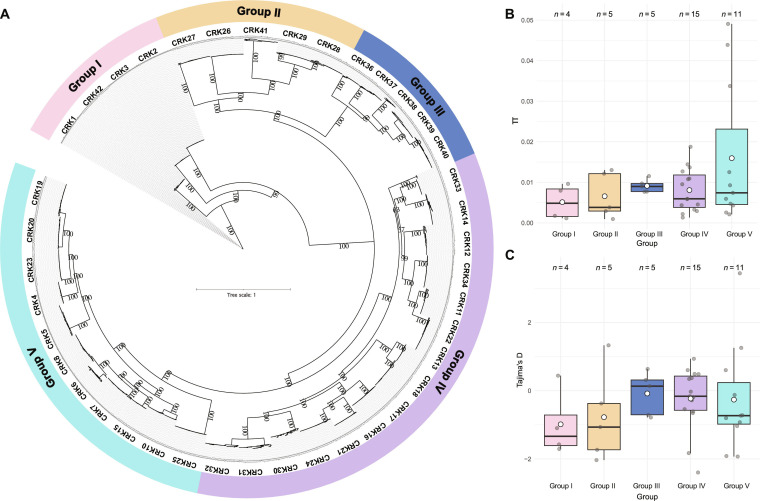
CRK phylogeny and natural genetic diversity. (A) Phylogenetic reconstruction based on the CRK coding sequence region across Arabidopsis ecotypes. Best model fit according to BIC score: TPM3u+F+I+R4. Numbers correspond to bootstrap values generated from 1,000 ultrafast bootstraps. (B) Nucleotide diversity (π) and (C) Tajima’s *D* per CRK grouped per phylogenetic group. White dots correspond to the mean, and light gray dots correspond to individual CRK members belonging to that group. Phylogenetic groups are represented in the *x*-axis; *n* corresponds to the number of CRKs per phylogenetic group.

Nucleotide diversity is a key feature to examine, as it offers insights into the evolutionary processes shaping genes—particularly those involved in vital functions such as development and defense responses [14,15]. Despite the CRK family in *A. thaliana* comprising 44 members, in our studies, we focused on 40 CRKs [2], as the excluded CRKs lacked either an ECD or a KD or appeared to be pseudogenes (Table S1 and Data S1). We assessed the natural genetic variation of CRKs across 69 naturally occurring *A. thaliana* ecotypes and obtained 2,760 CRK sequences corresponding to 40 CRK members (Data S2). Phylogenetic analysis of the evolutionary history of CRKs revealed that they cluster into the 5 major groups, independent of ecotype or sampling location of origin (Fig. 1A). The following groups were defined: Group I (CRK1, CRK2, CRK3, and CRK42); Group II (CRK26, CRK27, CRK28, CRK29, and CRK41); Group III (CRK36, CRK37, CRK38, CRK39, and CRK40); Group IV (CRK11, CRK12, CRK13, CRK14, CRK16, CRK17, CRK18, CRK21, CRK22, CRK24, CRK30, CRK31, CRK32, CRK33, and CRK34); and Group V (CRK4, CRK5, CRK6, CRK7, CRK8, CRK10, CRK15, CRK19, CRK20, CRK23, and CRK25). As previously reported by Vaattovaara et al. [9], Group I resembles the so-called basal clade. The genes encoding basal clade CRKs were shown to be spread across multiple chromosomes and likely evolved early, with orthologues present in all vascular plants. The remaining Groups II to V constitute the variable clade CRKs, and they are mostly clustered on Chromosome 4 in Arabidopsis and are less conserved, likely having diversified through the whole genome duplication events [9]. We examined these 5 CRK phylogenetic groups and analyzed the variation in genetic diversity and segregating alleles.

To understand the evolutionary history of CRKs, we estimated sequence-level measures of genetic diversity—π (nucleotide diversity) and Tajima’s *D*—for each CRK (Fig. 1B and C). The index π reflects the amount of nucleotide differences when pairwise comparing all sequences within a phylogenetic group, with a higher π indicating higher genetic diversity. Tajima’s *D* was used as an indicator of whether a sequence is evolving globally under neutral evolution (primarily stochastic accumulation of mutations) or nonneutral evolution (i.e., natural selection shaping the distribution of mutations). When Tajima’s *D* is above or below a value of +2 or −2, respectively, that indicates nonneutral evolution at the whole sequence level. We obtained π and Tajima’s *D* for each CRK and compared them across all 5 phylogenetic groups (Fig. 1B and C). Group V showed the highest levels of genetic diversity (π = 0.0178 ± 0.0190), followed by Group III (π = 0.0091 ± 0.0016), Group IV (π = 0.0081 ± 0.0052), and Group II (π = 0.0066 ± 0.0056), while Group I showed the lowest values (π = 0.0051 ± 0.0043). These π values indicate that CRKs accumulate genetic variation differently. For Tajima’s *D*, we found that the mean values across all groups were negative. Tajima’s *D* ranged from −0.984 in Group I to −0.081 in Group III. While negative values of Tajima’s *D* can reflect an excess of rare alleles, we cannot disentangle neutral or nonneutral evolution at the whole-sequence level, as no group showed values above +2 or below −2. However, when considering individual CRKs, CRK10 had a value of 3.44, while CRK26 and CRK24 had values of −2.03 and −2.40, respectively, indicating nonneutral evolution (Fig. S1). The values for genetic diversity and Tajima’s *D* that we found for the entire coding DNA sequence (CDS) region of CRKs are in the range of nucleotide-binding leucine-rich repeat (NLR) receptors in rice and *A. thaliana* [16,17].

To further explore the genetic variation in the CDS region, we identified haplotypes within each CRK group (Fig. S2). A haplotype corresponds to at least 2 CRKs having an identical amino acid sequence for the entire CDS region. In Group I, from a total of 276 sequences, 200 were distributed in 38 haplotypes (72%). In Group II, out of 339 sequences, 267 were placed in 41 haplotypes (72%); in Group III, out of 254 sequences, 172 were placed in 46 haplotypes (67%); in Group 4, out of 852 sequences, 684 were placed in 128 haplotypes (80%); and in Group V, a total of 504 sequences out of 623 were placed in 102 haplotypes (81%). The most common CDS haplotype among all CRKs was CRK24 from ecotype Abd-0, which was identical in 36 ecotypes. Taken together, the variation in the coding regions of CRKs and the grouping into haplotypes might reflect an interplay between abiotic and biotic factors distributed across different geographical regions.

### CRK domain structure and diversity

To examine the diversity of the CRK family members in more detail, we considered the amino acid composition and domain structure of CRKs (Fig. S3). The pairwise identity of the amino acid sequence of the full-length protein is higher than that of the ECD alone, indicating that the ECDs are more variable than the rest of the protein (Figs. S3B and S4). This highest diversity in the amino acid composition on the level of ECD can be explained by the general role of the ECD in other RK families in the perception of diverse signaling molecules and complex formation with other RKs [3].

Advances in structure-prediction software, such as the development of AF, enable reliable structural prediction of many proteins, including the individual domains of CRKs. The quality of AF predictions can be measured by the pLDDT (predicted local distance difference test) score. pLDDT scores can be ranked as >90 for very high confidence predictions, 70 to 90 for high confidence predictions, 70 to 50 for low confidence predictions, and <50 for very low confidence predictions. However, AF is unable to model full-length single-pass TM proteins (Fig. S5A to C) [18,19]. Thus, we focused solely on the ECD models in our analysis. AF models of CRK–ECDs (excluding the flexible juxtamembrane region) from the selected representatives of each phylogenetic group consistently yielded very high pLDDT scores (>90) and showed a similar fold for all CRK–ECDs, which suggests that AF accurately predicts the CRK–ECD structures (Fig. S5D). The predicted CRK–ECDs consist of 2 DUF26 domains. Each DUF26 domain has 2 α-helices connected to an antiparallel β-sheet containing 5 strands (Fig. S5D). The 2 DUF26 domains are connected by a flexible loop and through hydrophobic residues in their β-sheets.

To gain further insight into the putative function of the CRK family members, we analyzed in detail the predicted structures of the CRK–ECDs. We investigated homologs of CRK–ECDs to determine whether CRKs could function similarly. DUF26 domains are present in 2 other families of proteins: PDLPs and CRRSPs. The structures of 3 DUF26-containing proteins, the CRRSP Ginkbilobin-2 (GNK2), PDLP5, and PDLP8, have been experimentally resolved [9,20].

**Fig. 2.**
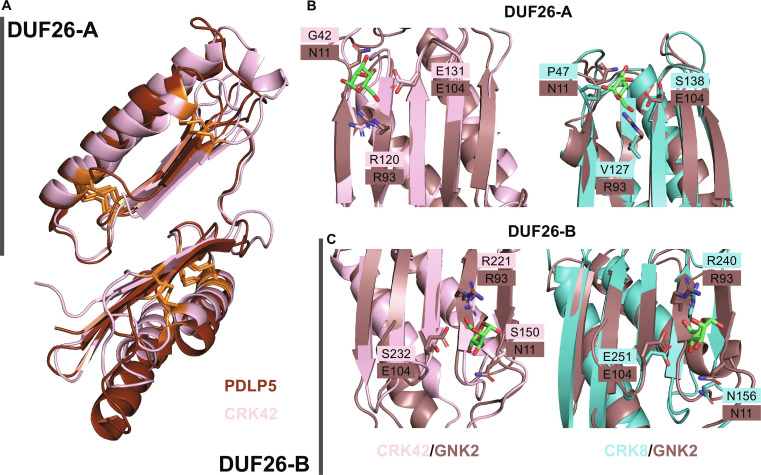
Structural alignments of predicted CRK–ECDs and their homologs. (A) Structure alignment of PDLP5 (brown, PDB: 6GRE [9]) to the predicted CRK42–ECD (pink, Group I). Group I CRKs are most similar to PDLPs, including 12 conserved cysteines (marked in orange). Structure alignment of GNK2 (brown, PDB: 4XRE [20]) to the predicted models of (B) CRK42–ECD (pink, Group I) and CRK8–ECD (teal, Group V) DUF26-A and (C) CRK42–ECD and CRK8–ECD DUF26-B. Mannose is indicated in green. The 3 residues involved in mannose-binding by GNK2 (N11, R93, and E104) and the respective AA in a similar position in the CRKs are represented as sticks.

PDLPs have an ECD consisting of a tandem of DUF26 domains and a TMD similar to CRKs, but they lack a KD. CRK–ECD AF models closely resemble the PDLP5 and PDLP8 crystal structures. PDLP-ECDs are the most similar to basal clade CRKs, and previous studies reported that PDLPs have likely evolved from basal clade CRKs (Fig. 2A) [9]. PDLP5 aligns to CRK42 with a root-mean-square deviation of 3 Å, indicating that the backbone atoms in the aligned structures are, on average, 3 Å apart. Both PDLPs and basal clade CRKs have 12 conserved cysteine residues forming 6 disulfide bonds, whereas variable clade CRKs display divergent cysteine conservation. PDLPs have no known ligand. However, they were shown to be involved in regulating callose deposition at the plasmodesmata [21].

GNK2 is a single DUF26 domain-containing protein from *Ginkgo biloba* with antifungal properties [20,22,23]. The structure of GNK2 has been resolved by both x-ray crystallography and nuclear magnetic resonance (NMR) spectroscopy [20,23]. Antifungal properties of GNK2 are most likely obtained due to its ability to bind mannose via residues N11, R93, and E104 [20] on the GNK2 β-sheet. Sequence alignments of GNK2 to CRK–ECDs show that GNK2 is most similar to the DUF26-A domain of Group I (basal clade) CRKs, and 2 of the mannose-binding residues are conserved (Fig. 2B and Fig. S6). However, for variable clade CRKs, no mannose-binding residues are conserved in DUF26-A. Interestingly, if GNK2 is aligned only with the DUF26-B domain of CRKs, it is shown that all 3 mannose-binding residues are conserved in Group V CRKs (Fig. 2C and Fig. S6). Aside from the 3 key mannose-binding residues, Miyakawa et al. [20] also identified 10 other residues of GNK2 (S8, A9, T12, Q13, C86, G91, A92, V94, Q105, and R106) by NMR, which were affected by mannose binding (chemical shift perturbation). However, these residues may also affect the binding site and are less conserved among CRKs (Fig. S6). Of these 10, A92 and V94 experienced the biggest chemical shift perturbation. A92 is conserved in 4 members of Group V and is substituted to G in the others. V94 is present in one Group V member and is substituted to L/I/T in 5 others. In addition, the presence of CRKs’ extra DUF26 domain may further influence the binding site.

Although the fold of all CRK–ECDs is similar, CRKs are expected to be involved in distinct signaling pathways, as are other RKs. By analyzing the diversity of amino acid sequences combined with the predicted structural data of CRK–ECDs, certain features were identified that may offer valuable insights into protein function. We generated amino acid sequence logo plots of the CRK–ECDs for each phylogenetic group, using the predicted structures of the representative CRKs as a reference, highlighting conserved and variable regions (Fig. S7). Conserved regions, such as the C-X8-C-X2-C motif, are hallmarks of this family of receptor kinases and are found across all phylogenetic groups. Additionally, each phylogenetic group has a unique set of conserved and variable regions, as shown in Fig. S7. In particular, those regions can indicate functional diversification.

Finally, we tested for signatures of positive and negative selection in representative CRK sequences from each group. We found signatures of both positive and negative selection (Fig. S8). In CRK39, we found a total of 34 amino acid sites under selection; in CRK29, 30; in CRK8, 30; in CRK42, 29; and in CRK11, 20. From the identified sites under selection, 47% (16 out of 34) were in the ECD region of CRK39, 50% (15 out of 30) were in the ECD region of CRK29, 37% (11 out of 29) were in the ECD region of CRK42, 25% (5 out of 20) were in the ECD region of CRK11, and 23% (7 out of 30) were in ECD region of CRK8. Together, these results highlight the contribution of different selective forces in shaping the natural genetic diversity of CRKs in *A. thaliana*. Considering the CRK–ECD structures for the analyzed representative CRKs, it is apparent that most of the residues under the selection pressure are located in the α-helices of either DUF26-A or -B. Fewer residues under selective pressure are located in β-sheet regions. Interestingly, there was a clearly higher contribution of the negative selection pressure. Negative selection acts on decreasing genetic variation that reduces fitness, for instance, removing mutations that would impair the sensing of pathogenic organisms or environmental cues. Some of these regions or residues contribute to the functional characteristics of the CRKs’ ECDs, including protein homology, glycosylation sites, cysteine residues, and probability for dimerization. In the following sections, we will explore these characteristics in greater detail.

### Glycosylation of the ECD is likely crucial for correct CRK expression and function

**Fig. 3.**
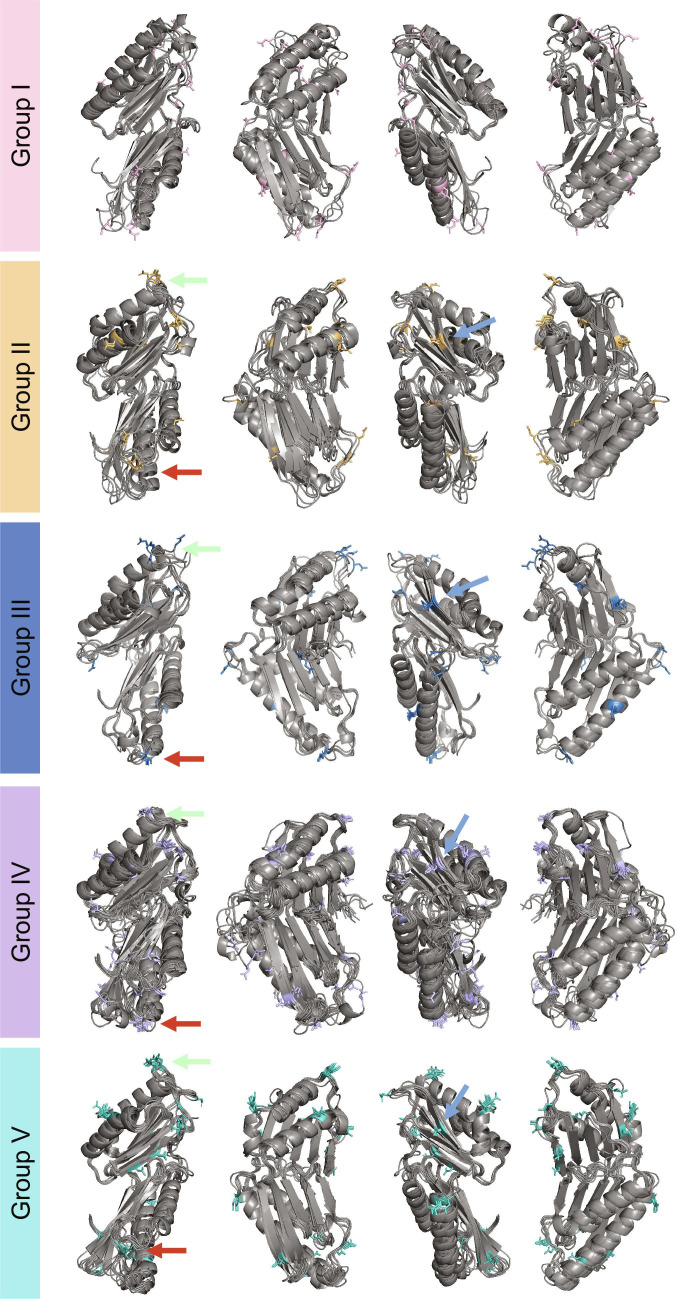
CRK–ECDs have multiple glycosylation sites. Predicted N-glycosylation sites of aligned CRK–ECDs per phylogenetic group based on the N-X-S/T motif (where X is not Pro). Asparagine residues that can be glycosylated are colored by phylogenetic group color. Three sites were identified where Group II to V CRKs have a conserved glycosylation site. Two sites are on loops of DUF26-A (green arrows) and DUF26-B (red arrows); the third site is on the edge β-strand of DUF26-A (blue arrows).

Glycosylation is essential for the folding and stability of proteins but can also be crucial for their function, interaction, and cellular processes’ localization [24–26]. de Oliveira et al. [27] showed that glycosylation of CRK4 and CRK5 was required for BAK1/SERK4-mediated cell death and suggested that glycosylation of CRKs is required for the folding of the protein, similar to other RLKs [26]. The type of sugar group and its attachment vary between glycosylation sites and organisms. Generally, 2 types of glycosylation are distinguished based on the attachment to the protein: N-glycosylation and O-glycosylation. O-glycosylation sites and the type of glycans attached cannot be predicted using the available predictive tools due to their complexity. However, potential N-glycosylation sites can be predicted for CRK–ECDs (Fig. 3). We performed these predictions for all the members of the 5 phylogenetic groups. When the CRK–ECD models are aligned within phylogenetic groups, 3 predicted N-glycosylation sites stand out, which are conserved among Group II to V CRKs (Fig. 3). These sites are present in relatively flexible loops of DUF26-A and DUF26-B (marked in green and red, respectively). Glycosylation at these positions might be important for protecting the most exposed part of DUF26 domains from, for example, proteolysis or oxidation. The third site (marked in blue) has a predicted glycosylation site on the side of DUF26-A and could potentially be used to shield the exposed edge β-strand, which can be prone to aggregation [28]. These conserved sites are not present in basal Group I, likely due to their different origin, but other glycosylation sites are predicted on loops and near edge β-strands.

### The potential functional role of cysteine residues in CRKs

One hallmark of CRK–ECDs is their number of cysteine residues (between 10 and 13), of which most are highly conserved (Figs. S4 and S7). When clustering CRK amino acid sequences by phylogenetic group, a distinct pattern of cysteine conservation emerges. In the basal clade (Group I), CRKs have 12 conserved cysteines. However, the variable clades (Groups II to V) diverge from this pattern and the number of cysteines. Notably, Group V entirely lacks the 2 cysteine residues at positions 167 and 252, while Groups II and IV lack the cysteine at position 167 and instead have 2 sequentially adjacent cysteines at positions 252 and 253 (Figs. S4 and S7). Lastly, Group III CRKs have several cysteine residues that are variably positioned. These variations in the number and positions of cysteine residues between the basal clade and the variable clades could suggest functional divergence.

**Fig. 4.**
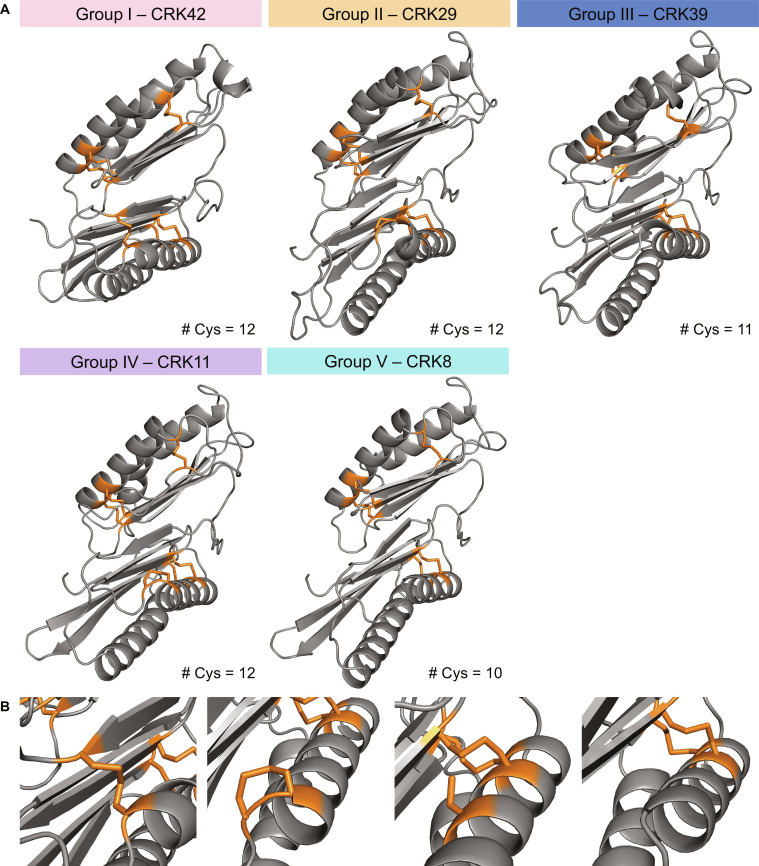
The distribution of the cysteine residues in CRK–ECDs. (A) Predicted models of representative CRKs for each phylogenetic group (CRK 42—Group I, CRK29—Group II, CRK39—Group III, CRK11—Group IV, and CRK8—Group V). The cysteines are marked in orange. The representative CRKs were chosen based on the highest average amino acid pairwise percentage identity of each CRK–ECD to the other members of the same phylogenetic group (Fig. S3). (B) The nonconserved cysteines are positioned on the first β-strand in DUF26-B of CRKs. There are 4 possible options in this position, from left to right: a disulfide bridge connecting secondary structural elements (Group I), a vicinal disulfide bridge (Groups II and IV), a single cysteine (Group III), or no cysteine (Group V).

Based on the protein crystal structure of PDLP5/PDLP8 and AF predictions, most cysteine residues in DUF26 domains are likely involved in disulfide bond formation (PDB: 6GRE and 6GRF) [9] (Fig. 4A). However, this does not apply to nonconserved cysteine residues. Compared to the basal clade (Group I), Group V CRKs lack a disulfide bridge, Group III CRKs often have an additional free cysteine, and CRKs in Groups II and IV have 2 sequentially adjacent cysteines (Fig. 4B). AF models suggest that the adjacent cysteines may form vicinal disulfide bonds. Vicinal disulfides are relatively rare and are often found in their reduced form or in disulfide bonds with other cysteines (instead of with each other) [29]. If the vicinal cysteines in CRKs do form a disulfide bond with each other, as is predicted by AF, then they could potentially have a functional role.

### Predicted mode of CRK–ECD dimerization

**Fig. 5.**
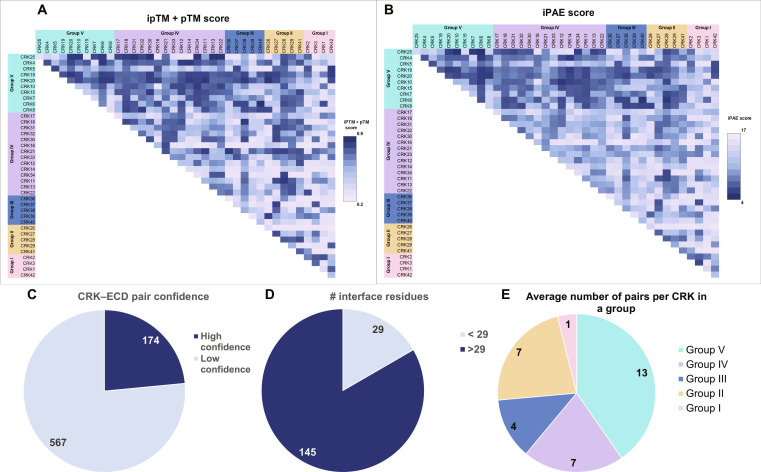
AF predicts the dimerization of CRKs. Heat maps of (A) ipTM + pTM and (B) iPAE scores for all CRK–CRK predictions. (C) The number of CRK–ECD pairs, out of all 741 pairs tested, that pass our ipTM + pTM, iPAE score cutoffs. (D) A pie chart representing the CRK–ECD pairs out of 174 that have more or less than 29 residues in the interaction interface suggesting high-confidence models. (E) Average number of CRK–ECD pairs that pass cutoffs per number of CRKs in a group.

A commonly reported mechanism for RK signal perception and transduction is dimerization of their ECDs. Few studies have reported CRK dimerization. Yadeta et al. [10] showed that CRK28 dimerizes with itself and with CRK29 at the full-length protein level. To establish a molecular basis for CRK interaction, we predicted CRK–CRK–ECD dimer models using AlphaFold2 [30]. Of the 40 CRKs, we used 38 CRK–ECD sequences as input, excluding CRK23, which has 3 DUF26 domains in its ECD, and CRK24, which only has 1. We predicted pairwise dimer models, generating 741 interaction pairs. For each CRK–ECD pair, 5 models were generated. The quality of these models was assessed using 2 metrics: the interface Predicted Template Modeling score and predicted Template Modeling score (ipTM + pTM), and the calculated “interface Predicted Aligned Error” (iPAE) score (Fig. 5A and B). The ipTM + pTM score is a metric that indicates the overall quality of the predicted structure, ranging from 0 for low-confidence predictions to 1 for high-confidence predictions [30]. The PAE is the expected error in the relative positions of residues toward each other, measured as the distance in angstroms (Å). For the iPAE score, we averaged the PAE scores across domains. For each pair, the best (out of 5) model that met our cutoffs (ipTM + pTM > 0.8 and iPAE < 6 Å) was selected, leaving 174 pairs as high confidence (Fig. 5C). We then used PDBePISA to further characterize the interface of all 174 remaining pairs, generating the number of interface residues per model in the pair (Data S3). Each interface had, on average, 34 ± 5.5 residues per model. A higher number of residues in the interface may indicate a stronger interaction, with more potentially interacting residues. We used the number of interface residues to set a new cutoff both models require 29 (average − 1 standard deviation) or more interface residues, leaving 145 (20%) of the initial CRK–ECD pairs (Fig. 5D and Table S2). Notably, the number of CRK–ECD pairs predicted with high confidence varies between phylogenetic groups (Fig. 5E).

**Fig. 6.**
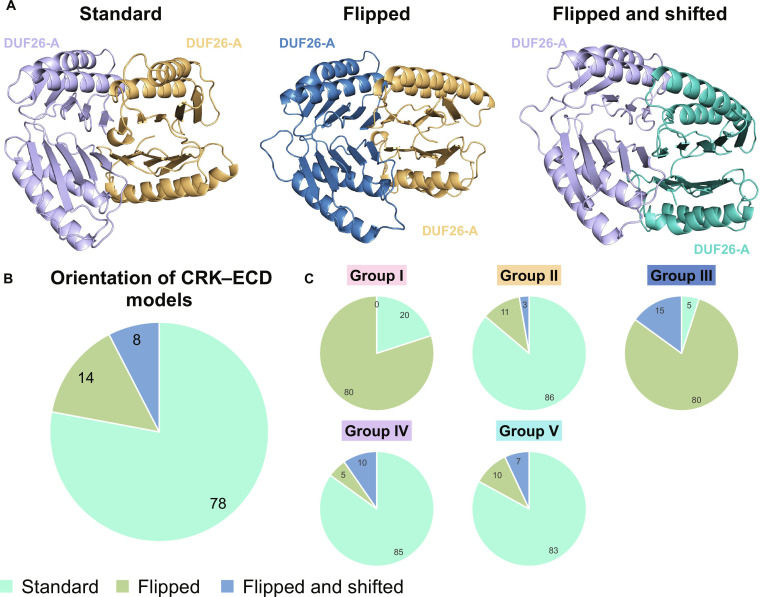
Orientation and distribution of predicted CRK–ECD dimers. (A) Three different classifications of orientations were identified, left to right: Standard, Flipped, and Flipped and shifted. The CRK pairs modeled are from left to right CRK31–CRK27 (Group IV and Group II), CRK38–CRK27 (Group III and Group II), and CRK11–CRK7 (Group IV and Group V). The DUF26-A position is marked, indicating the orientation of the ECD. (B) The percentage of each orientation that occurs in our datasets. CRK dimers are in the Standard orientation in 78% of the models. Other orientations occur less often. (C) Occurrence of each orientation per phylogenetic group. Per phylogenetic group, the dimer orientation of each dimer containing a CRK of that phylogenetic group was assessed. CRKs in Groups II, IV, and V all have a similar frequency at which the 3 orientations occur. Groups I and III have very few models in the Standard orientation.

Of the 145 CRK–ECD pairs selected, the ECDs may be oriented differently relative to each other. Three orientation models can be distinguished, which we named Standard, Flipped, and Flipped and Shifted (Fig. 6A). In 78% of high-confidence dimer pairs, the ECDs are in the Standard conformation (Fig. 6B). In this conformation, both ECDs are oriented so that the same side of each ECD interfaces with the other. The first β-strand of DUF26-B on each ECD forms the core of the dimerization interface. In the remaining 22% of pairs, the ECDs are in 1 of 2 other orientations (Flipped or Flipped and Shifted). In 14% of models, one ECD is flipped with respect to the other. In the Flipped orientation, DUF26-A of one ECD faces upward, while in the other, it faces downward, and the first β-strand of DUF26-A faces the first β-strand of DUF26-B of the other monomer. Finally, 8% of the models are in a Flipped and shifted orientation, where one ECD is flipped with respect to the other and shifted vertically, the same β-strand of DUF26-B of each ECD still interacts. If we analyze the distribution of the orientation types per member from each phylogenetic group, counting all dimers where one or both of the CRKs belong to the group, Groups I and III have few dimer predictions in the Standard orientation, while in Groups II, IV, and V, the majority of dimers are in the Standard orientation (Fig. 6C).

### The characteristics of the Standard interaction interface between ECDs

In 78% of the high-confidence CRK–ECD interaction pairs, the orientation of the ECDs with respect to each other is in the same Standard orientation, making it a likely candidate for a biologically relevant CRK–CRK dimerization mechanism. To gain further insight into the potential interaction mechanism, we evaluated the properties of the residues and the types of interactions involved in the dimerization interfaces of CRK–ECD pairs in this conformation.

**Fig. 7.**
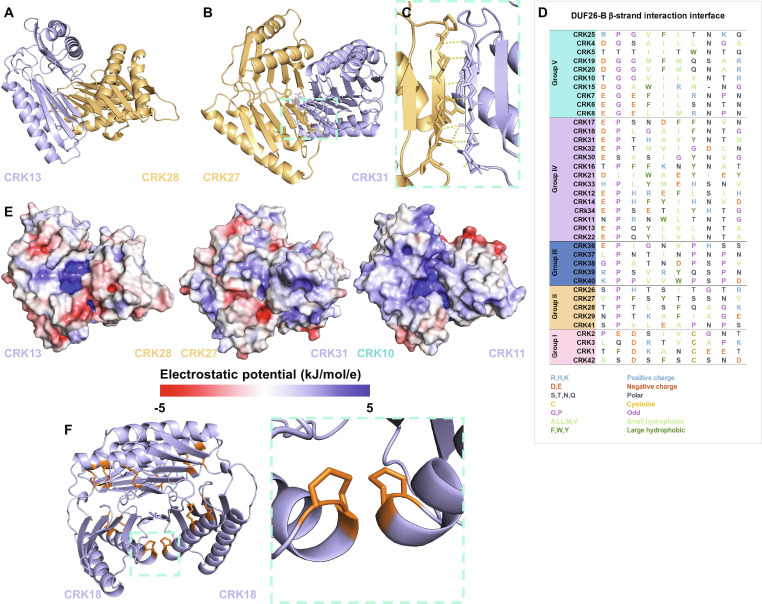
Properties of the various CRK–ECD interaction interfaces. (A) Seventy-two percent of CRK–ECD pairs have a predicted dimer interface on the β-strand of DUF26-B of both ECDs. In most Standard orientation models, the DUF26-A domains remain further apart. Dimer model shows CRK13–CRK28 (Group IV and Group II). (B) In a small subset, DUF26-A is also predicted to be in close proximity. Dimer model shows CRK27–CRK31 (Group II and Group IV). (C) Zoomed-in view of the DUF26-B β-strand interaction shown in (B). The β-strands mainly form hydrogen bonds through the amino acid backbone. (D) Amino acid sequence alignment of the interface β-strand. The residues in the interface are not conserved but are more hydrophobic. (E) Surface charge predictions of 3 models of CRK–ECD dimer pairs (CRK13–CRK28 [Group IV–Group II], CRK27–CRK31 [Group II–Group IV], and CRK10–CRK11 [Group V–Group IV]) show surface charge patches surrounding and inside the interface. Surface charge electrostatic potential ranges from −5 kJ/mol/e (red) to 5 kJ/mol/e (blue) [97]. APBS electrostatics was used in PyMOL to generate the maps. (F) CRK18–CRK18 pair (Group IV) showcasing the proximity of the vicinal disulfides to each other in the dimer. (Vicinal) disulfides are marked in orange.

In the Standard orientation, the DUF26-B domains in each ECD form an extended β-sheet together (Fig. 7A). In some interaction pairs, the DUF26-A domains of each CRK are also positioned close together, for example, in the CRK27–CRK31 dimer model (Fig. 7B). The core of the dimerization interface consists of hydrogen bonds between the first β-strand on DUF26-B of each CRK–ECD partner (Fig. 7C). Dimerization through β-sheet extension is common, as the interaction between β-strands from 2 distinct β-sheets can form similarly to those within sheets [28]. The residues in this β-strand are not conserved among CRKs, but generally, there are more hydrophobic residues present, which could be an additional stabilizing force during dimerization as interaction through hydrophobic patches is a common mechanism (Fig. 7D) [31]. An important factor in protein interaction is the number of contact points, such as hydrogen bonds and salt bridges, between the 2 molecules. Aside from the main interface through the DUF26-B β-strand, other regions might be involved. The number of contact points varies between CRK pairs and requires further characterization.

Martin-Ramirez et al. [2] reported that CRK–ECDs have diverse surface charges, which could contribute to the protein interaction. We selected 3 interaction pairs, CRK13–CRK28, CRK27–CRK31, and CRK10–CRK11, and modeled the surface charges of the dimers (Fig. 7E). In these examples, charged patches also surround the dimerization interface. In addition, in 2 of the interaction pairs, CRK13–CRK28 and CRK10–CRK11, highly charged patches are present in the interface between the ECDs, which could have a role in the dimerization interaction or ligand binding [32].

In the Standard orientation, the vicinal disulfides (present in CRKs from Groups II and IV) of each ECD can come in close proximity to each other, for example, in the CRK18–CRK18 homodimer (Fig. 7F). While the function of the vicinal disulfides in CRKs remains unknown, it is interesting that these potentially reactive residues are brought together by dimerization of CRKs. Taken together, the importance of β-strand interactions, surface charges, and vicinal disulfides for dimerization in CRK–ECDs in the Standard orientation provides interesting points for further investigation.

### CRK–ECDs form a highly interconnected predictive interaction network

**Fig. 8.**
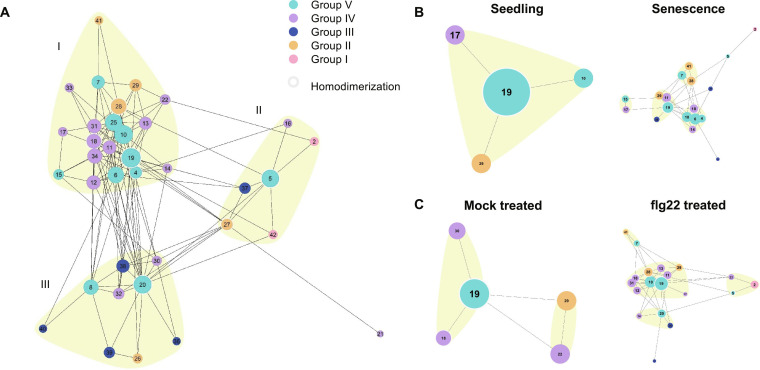
Interaction network of CRK–ECD AF predicted dimers. (A) The 145 AF CRK–ECD dimer predictions that passed our cutoffs were used to generate an interaction network. Each number represents a CRK–ECD, and the colored circles or nodes correspond to the phylogenetic group to which the CRK belongs. The node size corresponds to the number of interactions a CRK has. A light gray outline of the node indicates homodimerization. Lines connecting nodes indicate heterodimers. Communities of interconnected CRKs, as identified with the WalkTrap algorithm [33], are marked by a yellow background and are numbered I, II, and III. (B) Interaction networks refined using expression data of CRKs in seedlings and senescent plants from TravaDB [35]. (C) Interaction networks refined using expression data from Bjornson et al. [34] of CRKs in mock-treated seedlings or seedlings treated with 1 μM flg22 for 90 min.

Interacting receptors are components of complex signaling networks that initially perceive signals and ultimately trigger signal transduction, leading to appropriate cellular responses. To understand the dynamic range of intrafamily interactions between CRK–ECDs, network studies were used (Fig. 8A) [33]. In the network, each circle, known as a node, represents a CRK–ECD. The nodes are colored based on the phylogenetic group to which the CRK belongs. The lines, also known as edges, connect interacting CRK–ECDs and represent predictive physical interactions in this model. The size of the node is relative to the PageRank score, which serves as a centrality measure within the network, where larger node sizes indicate more interactions. Groups of CRKs with common interactors were clustered together into 3 communities (numbered I to III, highlighted by the yellow background) based on the WalkTrap algorithm. Lastly, homodimerization is marked by a gray circle surrounding the node. Homodimerization is predicted for 7 CRKs (CRK27, CRK28, CRK18, CRK4, CRK5, CRK19, and CRK20) (20% of the 38 CRKs tested).

In general, we observe a high number of connections between CRKs, leading to a dense network (Fig. 8A). The Group I CRKs form a few interactions that contribute to only one community, which could be a consequence of the different orientations of the ECDs in the dimer preventing interaction with variable clade CRKs. Similarly, the majority of models involving Group III CRKs display different dimer orientations and are involved only the smaller communities II and III. The core of the network primarily comprises CRKs from phylogenetic Groups II, IV, and V. Group V CRKs are predominantly represented within the network, consistent with previous observations that Group V CRKs have the highest number of interactions (Fig. 5E). Interestingly, Group V members CRK19 and CRK20, with a high sequence similarity (80% pairwise identity of their ECD), and a high number of interactions, are in different communities in the network. These findings suggest that CRK phylogenetic groups may play distinct roles within a larger connected network.

For CRKs to interact, they must be present within the same tissue type at an identical developmental stage. The networks were further refined utilizing CRK expression data across various developmental stages, tissue types, and stress treatments sourced from public databases, TravaDB, and Bjornson et al. [34,35]. A higher expression of CRKs is observed in senescent tissue relative to seedlings, as reflected in the interaction networks (Fig. 8B). Similarly, Arabidopsis seedlings exposed to 1 μM flg22 exhibit increased CRK expression, leading to a denser network (Fig. 8C). In both instances, heightened CRK expression is associated with an increase in ROS production due to either developmental processes, such as senescence, or defense responses triggered by flg22.

The majority of the CRKs found in the filtered networks are found in community I of the global network. It is very clear that seedling and mock-treated seedling networks have a low number of nodes (4 to 5), which is linked to a low expression of the CRKs at the seedling stage. Seedling and mock-treated seedling networks do not match completely, as they overlap only with CRK19 and CRK29. This could be explained by either biological and experimental variations, as these are 2 independent datasets or the effects of the mock treatment. While senescence and flg22-treated networks are similar in the number of CRKs (18 and 19 CRKs, respectively) and in the composition of the nodes, the distribution of individual nodes within smaller communities differs. This may indicate that a certain level of specificity is provided by the CRKs that are expressed and interact in response to different stimuli.

## Discussion

The computational predictions at the nucleotide and protein family levels greatly enhance our ability to infer function, understand biological processes, and efficiently guide experimental research. To gain evolutionary, biochemical, and structural understanding, we performed family-wide computational studies, which ultimately can support the experimental direction taken to unravel the biological roles of different CRK members. We surveyed the natural sequence variation, structural features, and predicted interaction landscape of the CRK family.

### Evolutionary dynamics and sequence diversity

The phylogeny identified 5 well-supported groups that mainly correspond to previous basal-versus-variable classifications but expands on this with group-specific diversity metrics [2,9] (Fig. 1A). Group V displayed the highest mean nucleotide diversity (π), whereas Group I (basal clade) was the least variable, consistent with an early origin and broader conservation across vascular plants [9] (Fig. 1B). The generally negative Tajima’s *D* values across groups indicate an excess of rare alleles, but only individual genes (CRK10, CRK24, and CRK26) showed Tajima’s *D* values consistent with deviation from neutrality, suggesting locus-specific departures due to recent selective sweeps, balancing selection, or demographic changes (Fig. 1C and Fig. S1). Interestingly, CRK10, which is known to play a role in the activation of defense responses in Arabidopsis, displays significantly higher Tajima’s *D* than other family members, consistent with balancing selection [36,37]. In this scenario, the alleles are maintained in the population, which is consistent with pathogen virulence genes evolving to evade host immune recognition. Some studies on Arabidopsis plants found low Tajima’s *D* values for receptor-like proteins (RLPs), similar to those for CRK24 and CRK26, suggesting purifying selection on genes involved in conserved developmental processes and the detection of conserved pathogen-associated molecular patterns (PAMPs) [38]. Haplotype distributions further confirmed that coding variation is unevenly distributed among groups, with some CDS haplotypes widespread across ecotypes (e.g., CRK24-Abd-0) and others more restricted (Fig. S2). This may be connected to geographically heterogeneous selective pressures or to genetic drift acting on locally adaptive variants [39]. Ngou et al. also found high levels of nucleotide diversity in genes encoding pattern-recognition receptors, indicating strong selective pressures and ongoing diversification, which are vital for adapting to various pathogen populations [40,41].

### ECD-centered structural diversification

Amino acid divergence is concentrated in ECDs, whereas the KD is highly conserved, implying that the intracellular signaling machinery is maintained while ligand recognition or interaction specificity has diversified [42] (Figs. S3, S4, and S7). ECDs of many RLKs bind ligands and interaction partners, making them important determinants of RK function [3,43]. To gain insight into the potential modes of interaction and functions of CRKs, we computationally investigated the properties of their ECDs. We could accurately predict the CRK–ECDs with AF, yielding models with structures displaying high similarity to the crystal structures of other DUF26-containing proteins, PDLP5, PDLP8, and GNK2 (Fig. 2). Despite the high degree of structural similarity, CRK–ECDs are characterized by multiple biophysical and biochemical features, including sequence diversity, surface charge, glycosylation sites, and number and distribution of cysteine residues, that differentiate them from PDLPs and GNK2 [2,9,44]. Importantly, many positively and negatively selected sites map to ECDs (23% to 50% of detected sites in representatives) (Fig. S8). The predominance of sites in which mutations are underflavored underscores the functional constraints on maintaining key structural elements, such as ligand-binding or interaction partner-binding interfaces. Similarly, in CLAVATA 1 (CLV1), nearly all residues were under negative selection. This was expected, as *clv1* mutants exhibit severe cell proliferation phenotypes across various species, supporting the idea that CLV1 is a conserved developmental gene subject to strong purifying selection [45]. However, CRKs also display localized positive selection, suggesting adaptive tuning of recognition surfaces, likely in response to diverse biotic and abiotic cues, and possibly related to ROS production. The work of Kileeg and Mott identified strong positive selection across many RLK gene subfamilies and a bias of positive selection in the ECDs of receptors [39,45,46]. RLKs involved in plant defense responses have signals of positive selection, particularly in regions responsible for pathogen recognition, indicating that these parts of the genes are evolving rapidly to enhance the plant’s immune capacity. This suggests that escape from adaptive conflict within the ECD may have played a substantial role in the evolution and adaptation of the RLKs. Moreover, positive selection was more common in the LRR domain than in other RLK domains [45,46]. This diversity in the signatures of selection suggests that CRKs may have distinct mechanisms for signal perception, complex formation, and modulation of different signaling pathways, which are essential for plant growth and defense.

### Functional implications of glycosylation and cysteine architecture

Distinct biochemical features can markedly influence the precise regulation of protein function. Therefore, we elected to conduct computational evaluations of some of these features at the family level. In plants, glycosylation is crucial for RLKs’ function, particularly for proper protein folding, trafficking, and stability [24]. It also directly regulates their activity, enabling responses to external stimuli by affecting ligand binding affinity and signal transduction. Key demonstrated roles include maintaining cell-surface receptor function in pattern-triggered immunity (PTI), like for the proper function of EF-TU RECEPTOR or developmentally guiding fertilization through pollen tube guidance mediated by FERONIA and its family members [24,47–49]. In the CRK family, conserved N-glycosylation motifs cluster in exposed flexible loops of DUF26-A and -B and on an exposed DUF26-A flank. Likely, these glycan moieties would plausibly stabilize ECD folding, protect labile loops from proteolysis/oxidation, and modulate interactions—consistent with prior experimental requirements for glycosylation in CRK function (Fig. 3).

Cysteine patterns differentiate basal and variable clades: basal CRKs preserve a 12-cysteine disulfide architecture, while variable clades show gains or losses, including vicinal cysteines (Fig. 4). These differences could alter local stability, create or remove redox-sensitive switches, or shape ligand-binding pockets. One hypothesis in the field is that CRK could sense ROS through its cysteine residues [5,50]. Vicinal disulfides have the potential to be redox active. In mammalian proteins such as von Willebrand factor and BMP-1, vicinal disulfides play key roles in protein function [51,52]. In these cases, the reduced state of the vicinal cysteines results in a more flexible, less stable protein, a feature crucial for regulating protein–protein interactions. However, Richardson et al. [29] analyzed existing vicinal disulfide-containing proteins in the PDB and showed that redox-active vicinal disulfides are not common. Instead, vicinal disulfides are often involved in ligand binding by providing a rigid hydrophobic contact point for sugars or multiring ligands [29]. The nonconserved cysteine residues in CRKs may play a functional role. However, if this function involves redox switching, is part of a binding pocket, or has a different role, it remains to be determined experimentally.

### Predicted mechanism of CRK–ECD interaction

Dimerization mechanisms of RLKs can give important insights into the involvement of the specific CRKs in the regulation of signaling pathways. To gain insight into the possible CRK–ECD dimerization mechanism, we studied the predictive dimerization specificity, dynamics, and the orientation of all predicted high-confidence dimers (Figs. 5 and 6). In 78% of high-confidence dimers, the CRK–ECDs are in the same orientation. The core of the identified interaction interface is an extended β-sheet formed by the interaction of the β-strands of DUF26-B domains. A similar extended β-sheet is also present in the Flipped and Flipped-and-Shifted orientation types. Vaattovaara et al. [9] proposed a similar dimerization mechanism based on the PDLP5 and PDLP8 crystal structure packing. The extended β-sheet forms an attractive model for a conserved DUF26 domain dimerization mechanism where the properties of the interface may determine specificity.

In general, β-sheets form through β-strand backbone hydrogen bonding. The edges of the β-sheet, also called β-edge strands, could interact further with other β-sheets if left exposed [28]. Proteins avoid unwanted interaction of β-edge strands through various mechanisms, including strands twisted by proline or glycine, charged residues, and short edge strands, or by covering edge strands with loops [53]. In order for β-strands to allow for dimerization, they require 5 or 6 exposed H-bonding residues that are regularly spaced [53]. CRK–ECDs have 4 edge strands. The first β-edge strand of DUF26-A is short and poorly predicted. The second edge strand is often partially covered by an α-helix. In addition, DUF26-A has conserved glycosylation sites near its β-edge strands. Glycosylation could further prevent the strands from interacting. The second DUF26-B β-strand is partially covered by a loop. The first β-edge strand of DUF26-B is the most exposed β-strand of CRK–ECDs and is part of the interface in AF predictions.

In addition to the β-strand interaction, the predicted dimers have notable charged regions near the interface (Fig. 7). One common mechanism for protein dimerization is through a surface characterized by a hydrophobic patch surrounded by charged residues [31,54].

### Dynamics of CRK–ECD interactions

Network analyses of the predicted high-confidence interactions among all CRK–ECDs reveal that the interacting proteins create a dense, highly interconnected network (Fig. 8). The communities identified are particularly rich in members of Groups II, IV, and V. Group V CRKs are especially important to the overall connectivity and structure of the networks, as indicated by their high average PageRank score. Among the predicted interacting CRK–ECDs, we found that they were previously shown to occur at the level of the full-length receptors’ homodimerization of CRK28 but not heterodimerization between CRK28 and CRK29 [10]. Community I has the highest number of interacting CRK–ECDs. Notably, many of them, such as CRK6, CRK7, CRK28, CK29, CRK10, and CRK14, have previously been reported to be involved in defense and abiotic stress responses [10,55–57]. This is very interesting and may suggest that stress-related CRKs cluster together, possibly forming receptor complexes responsible for stress response signaling.

The formation of these networks depends on CRKs being present in the same tissue and at the same developmental stage, as well as under specific stress conditions. Increased CRK expression during senescence or shortly after stress treatments such as flg22 exposure results in denser interaction networks. The networks also reveal that CRKs are more active and interconnected in certain tissues and under specific stimuli, suggesting functional specialization. Notably, seedling networks with low CRK expression show fewer interactions, while stress or developmental cues lead to complex, expansive networks. Differences in community distribution within similar networks suggest that CRKs may have specific roles depending on the stimulus or stage. Overall, the data emphasize the importance of both phylogenetic grouping and expression patterns in understanding CRK functions within plant signaling networks.

### CRKs as ROS or glycan receptors

Two types of signaling molecules have been hypothesized to be sensed by CRKs: ROS and glycans [2,58]. CRKs have been proposed as ROS receptors because of the high number of cysteines in their ECDs, which could undergo oxidative modification. ROS is produced in the apoplast during stress responses. While it is likely that ROS is also perceived in the apoplast as a signaling molecule, the RKs involved in ROS detection remain largely unknown. To date, only the leucine-rich repeat receptor kinase hydrogen peroxide-induced Ca^2+^ increases 1 (HPCA1) has been shown to be modulated by ROS in the apoplast [59]. CRKs have been linked to many defense responses that produce ROS [55,60,61]. It is also known that, transcriptionally, CRK members are up-regulated during developmental processes associated with elevated ROS levels or during various abiotic and biotic stresses [2]. However, a direct link between CRKs and ROS has never been demonstrated. Vaattovaara et al. [9] showed that in PDLP5 and PDLP8, all cysteines are involved in disulfide bonds, suggesting a role in the structural stability of DUF26 domains. However, this assessment does not include the nonconserved cysteines of CRKs. Cysteine residues involved in disulfide bridges are often required for protein stability and, as a result, conserved [62,63], suggesting that the nonconserved cysteines in variable clade CRKs may have a functional role rather than a structural one. In addition, the nonconserved cysteines in phylogenetic Groups II and IV are predicted to form vicinal disulfides, which are placed in close proximity in the dimer models. Willems et al. [63] showed that AF models of plant proteins correctly predict disulfide bonds. Vicinal disulfides are rare and can adopt unfavorable conformations, making them more likely to provide function outside of providing structural stability [29]. The vicinal cysteines in CRK–ECDs remain interesting candidates to study further. However, whether they function in a ROS sensing or in another capacity remains to be determined [64].

CRKs may function as glycan receptors due to their homology with GNK2 [20,22,23]. Glycans can originate from the plant or microbial cell wall and are important signaling molecules in both stress and developmental signaling processes [64,65]. GNK2 plays a role in fungal resistance through binding of mannose from the fungal cell wall [20]. Other DUF26-containing proteins from maize, AFP1 and AFP2, have also been implicated in glycan binding [66]. Additionally, a CRK from wheat, TaCRK3, also showed antifungal properties [67]. Sequence alignments of GNK2 to CRK DUF26 domains show that the mannose-binding motif is conserved in some Arabidopsis CRKs, making them interesting candidates for glycan binding. Recently, the work of Pierdzig et al. demonstrated that CRK7 functions as a receptor for bacterial-derived wall teichoic acids (WTAs) in Arabidopsis. When CRK7 encounters WTA, it forms homo-oligomers at the plasma membrane—a common mechanism for carbohydrate receptors—activating defense responses [68]. WTA is a much larger and more complex glycan than the mannose that GNK2 binds. Interestingly, CRK7 does have the 3 GNK2 mannose binding residues, but does not have all residues identified for GNK2 mannose binding. Whether CRK7 binds WTA through a similar mechanism as GNK2 binds mannose, and whether other CRKs may bind mannose or other glycans through a similar mechanism remains to be determined.

### The limitations of AF dimer predictions

AF offers a model for CRK–CRK ECD dimerization, but several limitations should be considered. AF is trained on experimentally resolved protein structures, and models could be biased toward it. This raises concerns about possible “memorization” from existing PDLP crystal structures.

A clear limitation of AF is its inability to model full-length CRKs; thus, it cannot assess if the TMD and KD contribute to the dimerization. Moreover, when modeling interactions of only the CRK–ECDs using AF, we cannot account for the effect of CRKs being anchored in the plasma membrane, limiting its mobility. However, the flexible extracellular juxtamembrane region, which has an average length of ~35 AA in CRKs, could allow CRK–ECDs a large range of motion, allowing them to be in different orientations that fit the predicted dimer configurations. In addition, the interaction orientations predicted by AF would only require each ECD to “bend” to the side with the juxtamembrane region extending from the last β-strand down to the membrane. Dimer predictions, while stringent (ipTM + pTM, iPAE, and interface residue thresholds), require biochemical and in planta validation: coimmunoprecipitation, Förster resonance energy transfer, crosslinking mass spectrometry, or cryo-EM/crystallography of ECD complexes.

In addition, the effects of the full-length protein on the CRK interaction and other factors, such as posttranslational modifications (glycosylation), pH, other interacting proteins, and ligands may influence CRK dimerization [3,69,70]. Glycosylation in particular could block access to the predicted interface. As noted above, the edge b-strand in the interface does not have predicted glycosylation sites in CRKs (Fig. 3). The recently released AF3 is a step closer to including these conditions, allowing for the prediction of glycosylation and small lipids [71]. However, it cannot overcome the lack of experimental data needed as input, for example, interaction partners or glycosylation sites and types of CRKs. While AF can provide valuable insights, it is not a replacement for experimental work [72].

### Future perspectives

Our integrated computational studies position CRK–ECDs as the principal component of diversification within the family, balancing strong purifying constraints on structural elements with localized adaptive changes in extracellular recognition surfaces. AF predictions give a likely mechanism for CRK–ECD dimerization, which can be used as a hypothesis to help guide future experiments to elucidate CRK interactions. Generally, the use of structural predictive tools and predicted structural data has increased considerably in recent years, enabling more effective analysis of protein structure and, in combination with experimental data, of cellular function [73,74]. Further developments in the structure prediction field, such as the recent release of AF3, can provide further insights by including posttranslational modifications and ligands in the predictions [71]. The predicted β-strand-mediated dimerization provides a mechanistic hypothesis for CRK complex assembly, with surface charge, glycosylation, and cysteine topology modulating interactions and responsiveness to environmental cues such as elicitor-triggered ROS or cell-wall-derived glycans. Moving forward, focused experimental tests are essential: (a) they validate key predicted homo- and heterodimers under native expression and membrane conditions; (b) they map glycosylation and redox states and assess effects on folding, localization, and complex formation; (c) they probe ligand-binding abilities of DUF26 variants (including mannose-like glycans) suggested by GNK2 homology; and (d) they relate allelic variation to phenotypic outcomes across ecotypes (disease resistance, ROS responses, and developmental regulation). Such work will clarify how CRKs incorporate evolutionary variation into plant signaling networks and adaptive responses.

## Methods

### Identification of CRK sequences across naturally occurring *A. thaliana* ecotypes

To identify the nucleotide sequences of CRKs across naturally occurring ecotypes of *A. thaliana*, we leveraged from the previously published chromosome-level pan-genome of 69 accessions [75]. We used the nucleotide sequences of all available CRKs in the TAIR database to search against the 69 whole genomes (Data S1). In brief, using makeblastdb (version 2.9.0) [76], we created local blast databases for each *A. thaliana* genome. Next, using the nucleotide sequences of each CRK as a query (Data S2), we performed a local blast search against each genome database. We extracted information on the start and end positions of the best blast hit using a custom R-script. With the identified region in blast, we extracted the nucleotide sequences from the target genome with samtools faidx (version 1.10) [77]. Finally, all sequences were concatenated into a single multisequence fasta file.

### Nucleotide sequence alignment and phylogenetic tree

The coding region of the extracted CRK nucleotide sequences was aligned with mafft (version 7.525) [78] using the “genafpair” option with a maximum of 1,000 iterations. From the resulting alignment, we used trimAl (version 1.5) [79] to exclude positions with more than 30% of gaps. The final alignment was used to reconstruct the phylogenetic tree with IQ-TREE2 (version 2.4.0) [80], setting the number of bootstrap replicates to 1,000 and automatic evolutionary model search. The final concatenated tree was visualized in iTOL [81].

### Natural selection, summary statistics, and ECD region diversity

For each CRK across haplotypes, we removed intronic regions, keeping only the amino acid coding regions (CDS). We generated haplotype networks using the TCS method implemented in the PopART package [82,83]. Additionally, using the PopART package, we determined the frequencies of haplotypes within each CRK. Nucleotide diversity (π) and Tajima’s *D* were computed using the R-packages PopGenome (version 2.7.7) and pegas (version 1.3). We used the HyPhy package [84] to search for signatures of natural selection considering 3 underlying models of evolution: mixed effect model of evolution [85], fast unconstrained Bayesian approximation [86], and single-likelihood ancestor counting [87]. AliView (version 1.26) [88] was used to translate the nucleotide coding region of our CRK clade groups into amino acids. After retaining only the ECD, we generated logo plots using the Python module Logomaker (version 0.8.6) [89].

### Sequence alignments

Amino acid sequences of Arabidopsis CRKs were retrieved from TAIR. The pairwise identity matrix and multiple sequence alignments were generated using Clustal-O (version 1.2.4) [90,91] and Muscle (version 3.8) [92], respectively, using the EMBL-EBI job dispatcher [93].

### AF modeling

Full-length CRK models were extracted from the AF database [13,94]. All other AF predictions were performed using a local installation of AF v2.2.2. Parameters were set to default; 5 random seeds were generated with one model per seed. For monomer ECD models, the amino acid sequence from the first residue to the predicted start of the TMD was used as input. The highest scoring models were used for the figures, and N- and C-terminal flexible regions with a low pLDDT confidence score were removed.

To model the CRK–ECD dimers, we cropped the input sequence to contain only the structured DUF26 domains, as low confidence flexible N- and C-terminal regions could interfere with the predictions. Dimer models were predicted for 780 pairs, generating 5 models each. The ipTM + pTM scores were generated by AF. PAE scores were generated by AlphaPickle, and the interface specific scores (iPAE) were extracted [95]. Per CRK–ECD pair, the highest-scoring model that had an ipTM + pTM score and an iPAE score above 0.8 and an iPAE score smaller than 6 Å was selected as a high-confidence prediction. Next, for each model passing ipTM + pTM and iPAE scores, the number of interface residues was determined using PDBe PISA [96]; models with 29 (average − 1 standard deviation) or more residues in each CRK–ECD interface were selected. The 145 CRK–ECD pairs that were modeled with high confidence were further inspected manually. Model figures were prepared in PyMOL. Surface charge maps were generated using the Adaptive Poisson-Boltzmann Solver software in PyMOL using standard settings (pH 7, 0.15 M ionic strength) [97].

### Network construction and analysis

The network was constructed using the igraph package (http://igraph.org/r/) in the R programming environment (https://www.r-project.org/). Clusters of interacting proteins in the network were identified using the WalkTrap algorithm as implemented in igraph using a random walk length of 8 (reference DOI: 10.1038/srep05739) [33]. The PageRank algorithm implementation using the PRPACK library within the igraph package was used as a centrality measure within the network, with the node size set to be relative to the PageRank score.

## Data Availability

AF dimer models can be found on Zenodo at DOI: 10.5281/zenodo.18742569.
