# Supplementary material for: Genetic Diversity, Predictive Protein Structures, and Interaction Networks of Cysteine-Rich Receptor-Like Kinases in Arabidopsis thaliana
Source: Comput Struct Biotechnol J. 2026 Apr 8;35(1):0043. doi: 10.34133/csbj.0043 (PMC13058244; doi:10.34133/csbj.0043)
Supplement: Supplementary 1 — Figs. S1 to S8 Tables S1 and S2 Data S1 to S3 [file csbj.0043.f1.zip › SupplementaryFigure3.pdf]

A

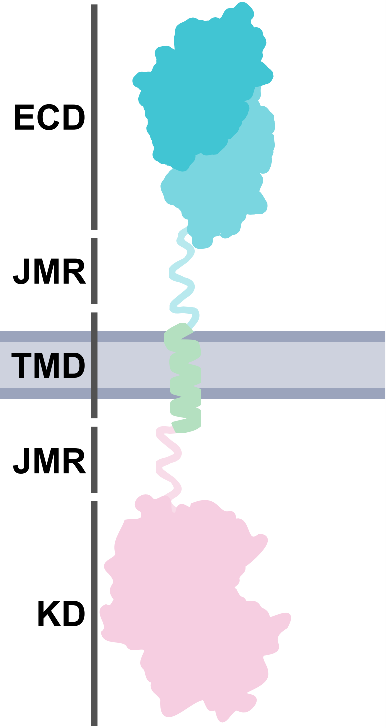

B

Pairwise identity of full length CRK sequences

Row axis: Pairwise identity of CRK ECD sequences

		Group V										Group IV														Group III					Group II					Group I				
		CRK25	CRK4	CRK5	CRK19	CRK20	CRK10	CRK15	CRK7	CRK6	CRK8	CRK17	CRK18	CRK31	CRK32	CRK30	CRK16	CRK21	CRK33	CRK12	CRK14	CRK34	CRK11	CRK13	CRK22	CRK36	CRK37	CRK38	CRK39	CRK40	CRK26	CRK27	CRK28	CRK29	CRK41	CRK2	CRK3	CRK1	CRK42	Average per group
Group V	CRK25		55	54	55	55	57	57	53	54	53	45	47	44	44	44	46	45	46	49	49	50	48	42	44	41	43	39	40	39	45	43	41	42	40	32	32	31	31	50
	CRK4	52		64	71	70	62	62	60	59	60	48	48	46	46	47	49	45	45	51	47	51	50	41	45	40	41	40	38	40	43	41	41	42	41	32	32	30	33	62
	CRK5	46	52		66	67	59	55	56	54	54	48	48	48	48	48	50	45	45	51	47	50	49	43	45	40	41	41	39	40	43	42	40	40	40	32	32	29	34	47
	CRK19	47	61	46		84	64	60	60	61	60	49	50	47	48	49	51	47	46	52	49	52	50	43	46	41	41	41	39	40	44	43	42	42	42	33	33	28	33	58
	CRK20	49	64	48	80		65	63	62	63	64	48	49	48	48	49	50	47	46	51	50	53	50	44	45	41	41	41	39	40	43	43	41	42	40	32	32	29	33	61
	CRK10	51	63	49	61	63		68	69	69	68	49	50	47	47	48	48	46	48	50	49	52	50	44	46	43	42	40	40	41	46	44	42	44	42	34	32	30	33	59
	CRK15	56	67	47	54	58	57		71	71	72	48	48	46	46	47	47	45	45	51	49	50	50	45	46	40	41	39	40	41	44	42	43	44	41	35	35	31	35	59
	CRK7	50	64	45	57	62	62	63		74	76	47	46	46	46	46	47	45	45	49	49	51	50	44	45	39	41	39	38	39	42	43	43	43	41	32	33	30	33	62
	CRK6	51	67	44	58	63	61	65	73		86	48	48	46	46	46	47	45	46	50	49	50	49	44	45	40	40	39	39	40	44	43	41	42	41	32	32	30	34	62
	CRK8	50	69	47	59	64	62	67	79	72		49	48	47	46	46	48	44	45	50	49	50	48	43	43	40	40	38	38	39	44	42	41	43	42	33	33	31	34	63
Group IV	CRK17	29	23	24	26	25	25	26	25	28	29		76	56	56	56	54	53	51	57	56	55	54	51	52	42	42	41	44	44	40	41	40	41	41	33	31	31	31	43
	CRK18	29	24	23	24	25	24	24	23	26	25	56		57	56	59	56	56	50	58	56	56	56	51	52	42	43	41	43	42	41	42	39	39	40	32	32	31	32	45
	CRK31	28	23	22	22	23	23	21	25	23	25	43	45		82	58	56	56	49	51	54	56	55	50	52	44	44	40	43	43	41	41	41	41	39	33	33	32	34	45
	CRK32	25	22	20	24	23	23	21	25	22	24	39	41	72		57	56	56	50	52	55	57	56	49	52	43	42	40	42	42	40	40	40	40	38	34	32	31	33	44
	CRK30	30	24	21	26	29	26	25	27	26	25	40	46	42	41		58	57	52	55	58	55	55	48	52	40	43	41	41	41	41	41	40	39	39	32	31	30	30	44
	CRK16	28	27	26	26	29	28	25	29	24	29	40	40	41	42	43		61	50	54	56	53	53	49	48	42	42	41	41	40	42	41	41	40	39	31	32	31	32	41
	CRK21	28	23	22	25	26	26	23	24	25	25	32	37	35	34	37	37		50	50	53	53	53	47	48	41	42	41	42	41	39	41	38	38	37	33	31	32	32	37
	CRK33	31	23	23	24	26	27	24	27	27	28	38	37	39	38	44	40	34		53	56	56	55	51	51	43	44	41	44	42	40	42	41	40	39	32	31	28	31	42
	CRK12	34	29	27	29	28	26	30	29	29	30	42	44	36	37	41	38	33	41		62	59	55	54	52	41	42	41	44	42	40	42	41	40	41	32	31	30	32	42
	CRK14	35	28	28	31	33	31	32	32	31	32	50	49	48	47	49	51	40	49	49		60	58	53	54	42	42	42	45	43	41	43	42	41	41	33	32	30	32	49
	CRk34	35	28	27	29	30	28	28	29	27	29	47	46	43	42	46	42	40	45	48	53		61	53	57	43	43	44	46	44	42	44	43	43	41	32	31	30	33	46
	CRK11	33	26	25	27	29	28	26	31	30	30	48	51	49	49	50	44	43	47	45	55	53		57	68	43	44	40	44	44	43	43	41	42	40	32	32	29	33	54
	CRK13	33	24	23	23	24	26	24	27	26	25	45	46	47	47	46	40	38	46	44	50	48	61		70	39	40	38	40	41	38	39	38	39	39	32	31	29	31	51
	CRK22	32	23	24	23	24	26	24	27	26	25	45	46	47	47	46	38	37	45	43	48	47	61	97		40	39	39	41	41	40	40	38	39	38	32	30	28	30	50
Group III	CRK36	31	24	26	29	28	31	24	26	26	26	32	32	33	33	31	33	29	33	34	39	34	37	36	36		58	56	60	58	41	42	42	42	41	32	31	29	32	44
	CRK37	29	26	24	25	25	28	25	30	27	28	31	30	33	30	34	36	33	35	36	38	35	39	32	32	45		59	59	58	40	42	41	40	40	30	31	27	31	50
	CRK38	25	24	22	23	22	24	24	23	24	23	29	29	26	26	27	28	27	29	30	32	33	28	28	28	40	50		62	60	39	42	42	40	40	29	31	29	30	49
	CRK39	31	23	21	26	25	27	26	25	26	25	35	36	35	32	31	32	31	34	35	42	38	35	35	35	49	55	57		80	39	40	41	40	40	32	30	28	31	55
	CRK40	28	26	22	26	25	27	27	27	26	26	35	33	33	32	32	30	26	31	32	37	36	35	35	35	43	48	49	60		38	40	41	41	40	33	32	29	32	50
Group II	CRK26	37	33	31	34	33	36	29	31	32	33	26	27	26	23	29	30	24	28	27	30	30	31	29	29	26	25	22	24	22		50	47	48	46	35	33	31	32	39
	CRK27	34	27	27	29	31	28	27	28	29	27	27	29	27	24	32	30	28	28	29	36	29	32	31	31	29	30	28	28	24	42		47	47	45	33	32	32	33	38
	CRK28	33	31	28	33	32	30	28	31	29	29	28	25	24	24	29	29	25	26	28	32	29	28	29	28	30	27	27	27	28	37	39		81	54	31	34	32	32	52
	CRK29	34	34	29	34	34	32	29	33	31	31	28	25	26	26	30	30	26	27	30	32	29	30	29	30	29	27	26	26	27	42	39	80		54	31	34	32	32	53
	CRK41	28	30	25	27	29	27	29	31	28	31	28	25	22	20	26	26	22	26	30	30	25	26	27	26	27	26	24	24	26	36	32	50	50		32	32	30	29	42
Group I	CRK2	22	22	22	23	22	23	24	19	19	21	20	17	18	20	20	18	19	18	20	21	18	18	20	20	18	17	16	18	21	22	19	19	18	17		42	41	42	34
	CRK3	22	20	21	20	20	18	23	20	18	22	19	21	20	20	22	22	17	19	20	24	19	20	19	19	17	15	16	15	18	19	17	20	21	20	34		41	45	32
	CRK1	22	20	19	17	18	17	18	17	17	18	14	15	16	14	18	19	16	16	16	18	16	17	16	16	16	16	15	11	14	18	22	21	20	18	29	29		45	31
	CRK42	22	24	23	21	22	21	24	20	21	21	15	18	21	21	18	20	19	20	19	22	19	20	20	20	20	17	17	18	20	20	21	22	20	18	38	32	34		35
