# Supplementary material for: Genetic Diversity, Predictive Protein Structures, and Interaction Networks of Cysteine-Rich Receptor-Like Kinases in Arabidopsis thaliana
Source: Comput Struct Biotechnol J. 2026 Apr 8;35(1):0043. doi: 10.34133/csbj.0043 (PMC13058244; doi:10.34133/csbj.0043)
Supplement: Supplementary 1 — Figs. S1 to S8 Tables S1 and S2 Data S1 to S3 [file csbj.0043.f1.zip › SupplementaryFigure6.pdf]

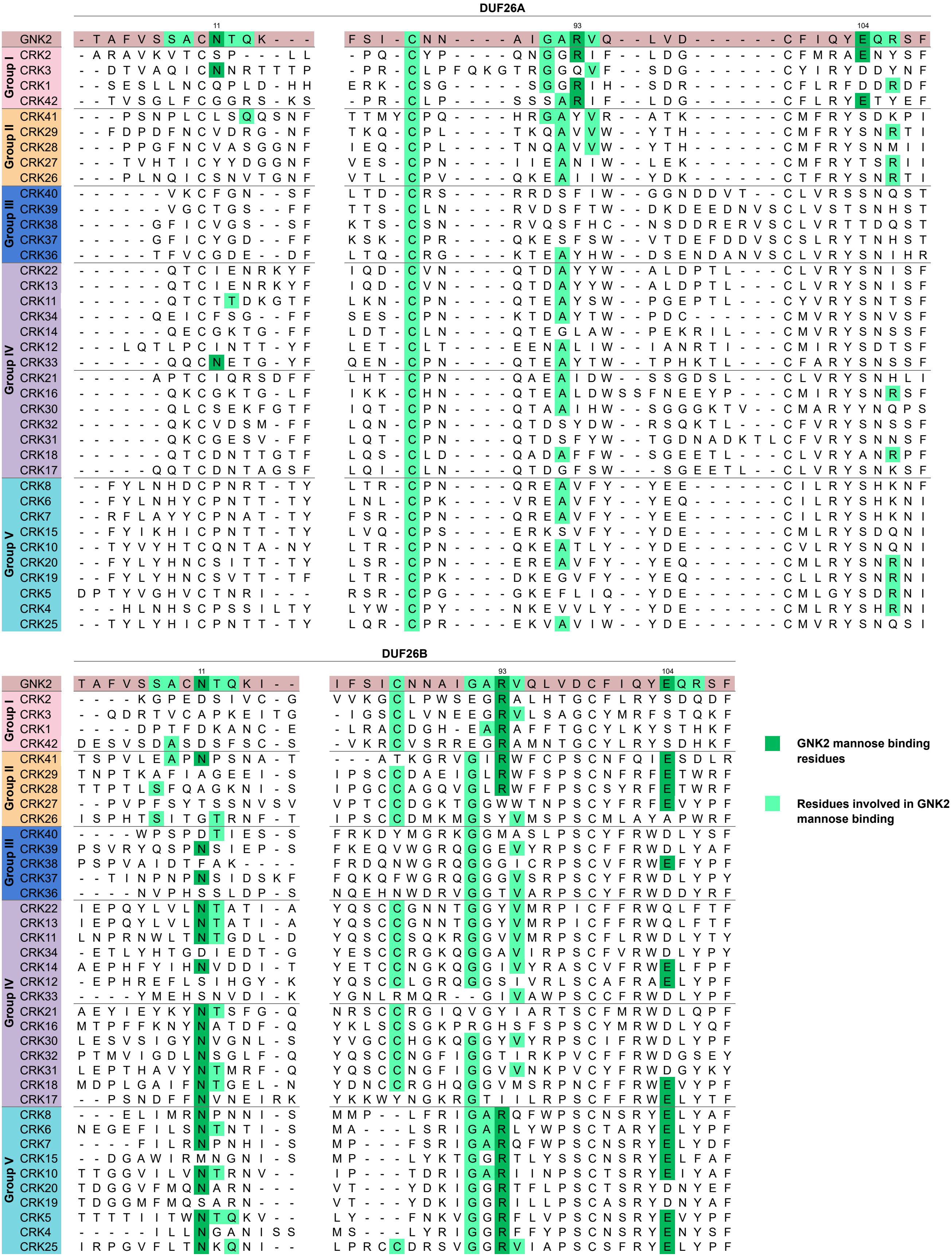
DUF26A
DUF26B
GNK2
Group I
Group II
Group III
Group IV
Group V
11
93
104
GNK2 mannose binding residues
Residues involved in GNK2 mannose binding
