# Supplementary material for: Genetic Diversity, Predictive Protein Structures, and Interaction Networks of Cysteine-Rich Receptor-Like Kinases in Arabidopsis thaliana
Source: Comput Struct Biotechnol J. 2026 Apr 8;35(1):0043. doi: 10.34133/csbj.0043 (PMC13058244; doi:10.34133/csbj.0043)
Supplement: Supplementary 1 — Figs. S1 to S8 Tables S1 and S2 Data S1 to S3 [file csbj.0043.f1.zip › SupplementaryTable1.pdf]

CRK	uniprot_ID	TAIR	TAIR_representative	gene_length	strand	synteny
CRK1	Q9LMB9	AT1G19090	AT1G19090.1	2266	positive	solo
CRK2	Q9CAL3	AT1G70520	AT1G70520.1	2447	negative	clusterG
CRK3	Q9CAL2	AT1G70530	AT1G70530.1	2630	negative	clusterG
CRK4	Q9LZU4	AT3G45860	AT3G45860.1	2641	negative	solo
CRK5	Q9C5S8	AT4G23130	At4g23130.2	2447	negative	solo
CRK6	Q9C5S9	AT4G23140	At4g23140.2	2641	positive	solo
CRK7	Q8L7G3	AT4G23150	AT4G23150.1	2571	positive	solo
CRK8	O65468	AT4G23160	AT4G23160.2	2762	positive	solo
CRK10	Q8GYA4	AT4G23180	AT4G23180.1	2610	positive	clusterA
CRK11	Q9ZP16	AT4G23190	AT4G23190.1	2514	negative	clusterA
CRK12	O65472	AT4G23200	AT4G23200.1	2555	negative	clusterB
CRK13	Q0PW40	AT4G23210	AT4G23210.3	2527	negative	clusterB
CRK14	Q8H199	AT4G23220	AT4G23220.1	2791	negative	clusterC
CRK15	Q8W4G6	AT4G23230	AT4G23230.1	2453	negative	clusterC
CRK16	O65476	AT4G23240	AT4G23240.1	2692	negative	clusterC
CRK17	Q8L710	AT4G23250	AT4G23250	2576	negative	clusterC
CRK18	Q8RX80	AT4G23260	AT4G23260.1	2528	negative	clusterC
CRK19	Q8GWJ7	AT4G23270	AT4G23270	2913	negative	clusterC
CRK20	O65479	AT4G23280	AT4G23280	2732	positive	clusterC
CRK21	Q3E9X6	AT4G23290	At4g23290.2	2901	negative	clusterC
CRK22	Q6NQ87	AT4G23300	AT4G23300.1	2530	positive	clusterC
CRK23	O65482	AT4G23310	AT4G23310.1	3027	positive	clusterC
CRK24	O65483	AT4G23320	AT4G23320	2109	negative	clusterC
CRK25	Q9M0X5	AT4G05200	AT4G05200	2517	negative	solo
CRK26	Q9T0J1	AT4G38830	AT4G38830.1	2605	positive	solo
CRK27	O49564	AT4G21230	AT4G21230.1	2436	negative	solo
CRK28	O65405	AT4G21400	AT4G21400.1	2492	negative	clusterD
CRK29	Q8S9L6	AT4G21410	AT4G21410	2188	negative	clusterD
CRK30	Q9LDT0	AT4G11460	AT4G11460.1	2626	positive	clusterE
CRK31	Q9LDM5	AT4G11470	AT4G11470	2433	positive	clusterE
CRK32	Q9LDS6	AT4G11480	AT4G11480.1	2392	positive	clusterE
