# Supplementary material for: Genetic Diversity, Predictive Protein Structures, and Interaction Networks of Cysteine-Rich Receptor-Like Kinases in Arabidopsis thaliana
Source: Comput Struct Biotechnol J. 2026 Apr 8;35(1):0043. doi: 10.34133/csbj.0043 (PMC13058244; doi:10.34133/csbj.0043)
Supplement: Supplementary 1 — Figs. S1 to S8 Tables S1 and S2 Data S1 to S3 [file csbj.0043.f1.zip › SupplementaryTable2.pdf]

# CRK-ECDs:					Orientation:					
	#	%				#	%			
Tested	38				Standard	113	78			
Pairs	741				Flipped	21	14			
Pass cut-offs	145	20			Flipped and shifted	11	8			

Per phylogenetic group:

Group	# CRKs	# Interactions	Average		Standard	%	Flipped	%	Flipped and shifted	%
Group V	10	129	13		107	83	13	10	9	7
Group IV	14	93	7		79	85	5	5	9	10
Group III	5	20	4		1	5	16	80	3	15
Group II	5	36	7		31	86	4	11	1	3
Group I	4	5	1		1	20	4	80	0	0

Per CRK:

CRK	# Interactions	% of total	Homodimer		Standard	%	Flipped	%	Flipped and shifted	%
CRK25	14	37	0		14	100	0	0	0	0
CRK4	11	29	1		9	82	0	0	2	18
CRK5	8	21	1		5	63	2	25	1	13
CRK19	24	63	1		20	83	3	13	1	4
CRK20	21	55	1		18	86	2	10	1	5
CRK10	14	37	0		14	100	0	0	0	0
CRK15	5	13	0		5	100	0	0	0	0
CRK7	7	18	0		5	71	1	14	1	14
CRK6	14	37	0		11	79	3	21	0	0
CRK8	11	29	0		6	55	2	18	3	27
CRK17	3	8	0		3	100	0	0	0	0
CRK18	14	37	1		11	79	1	7	2	14
CRK31	10	26	0		10	100	0	0	0	0
CRK32	7	18	0		6	86	1	14	0	0
CRK30	6	16	0		5	83	1	17	0	0
CRK16	2	5	0		1	50	0	0	1	50
CRK21	1	3	0		1	100	0	0	0	0
CRK33	3	8	0		3	100	0	0	0	0
CRK12	10	26	0		7	70	0	0	3	30
CRK14	4	11	0		4	100	0	0	0	0
CRK34	10	26	0		9	90	0	0	1	10
CRK11	11	29	0		8	73	1	9	2	18
CRK13	7	18	0		7	100	0	0	0	0
CRK22	5	13	0		4	80	1	20	0	0
CRK36	2	5	0		0	0	0	0	2	100
CRK37	4	11	0		0	0	4	100	0	0
CRK38	9	24	0		0	0	9	100	0	0
CRK39	3	8	0		1	33	1	33	1	33
CRK40	2	5	0		0	0	2	100	0	0
CRK26	3	8	0		2	67	1	33	0	0
CRK27	11	29	1		9	82	2	18	0	0
CRK28	14	37	1		13	93	0	0	1	7
CRK29	6	16	0		6	100	0	0	0	0
CRK41	2	5	0		1	50	1	50	0	0
CRK2	2	5	0		1	50	1	50	0	0
CRK3	0	0	0		0	0	0	0	0	0
CRK1	0	0	0		0	0	0	0	0	0
CRK42	3	8	0		0	0	3	100	0	0
