# Supplementary figures and images for: Genetic Diversity, Predictive Protein Structures, and Interaction Networks of Cysteine-Rich Receptor-Like Kinases in Arabidopsis thaliana
Source: Comput Struct Biotechnol J. 2026 Apr 8;35(1):0043. doi: 10.34133/csbj.0043 (PMC13058244; doi:10.34133/csbj.0043)

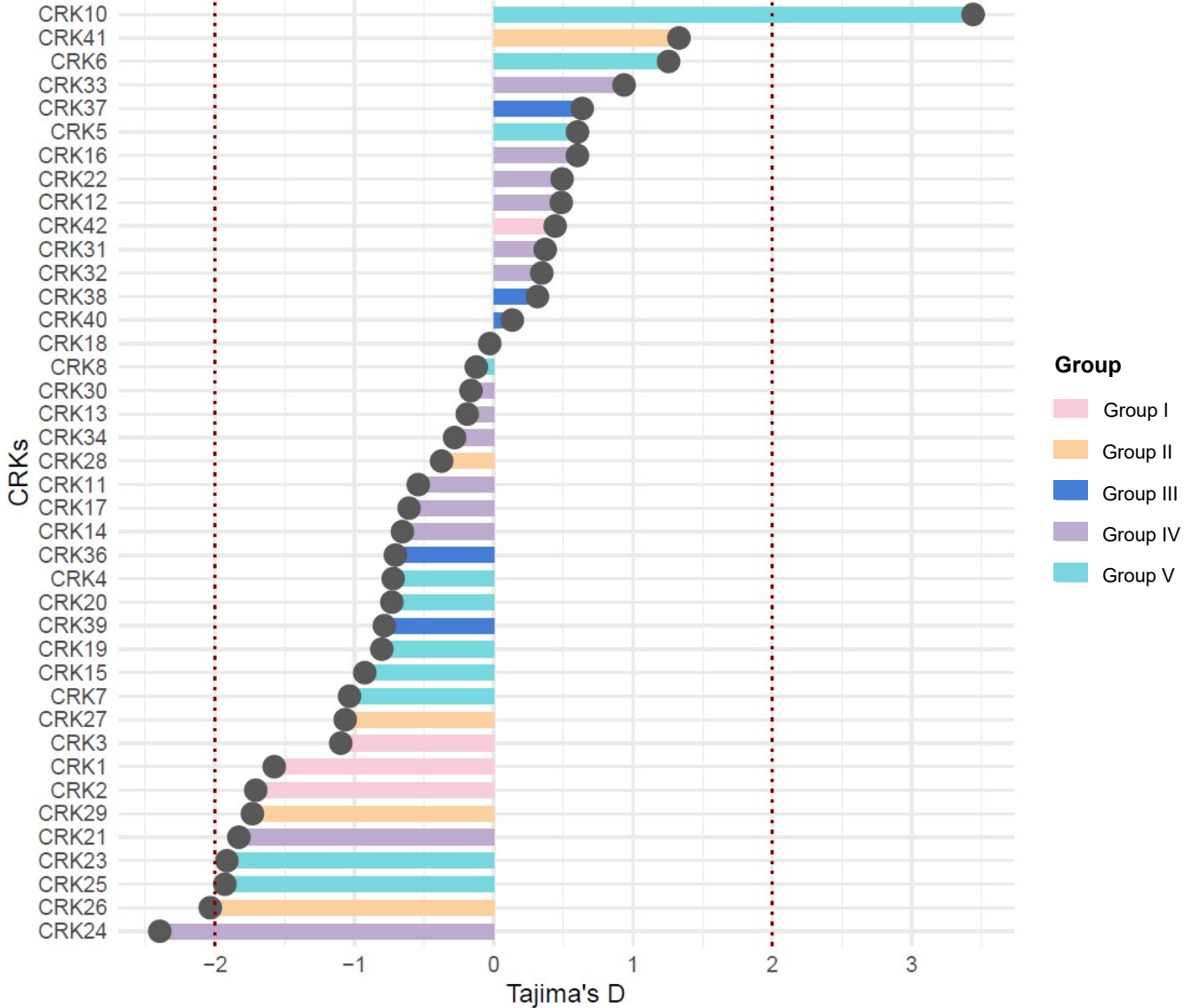

Supplement: Supplementary 1 — Figs. S1 to S8 Tables S1 and S2 Data S1 to S3 [file csbj.0043.f1.zip › SupplementaryFigure1.pdf]

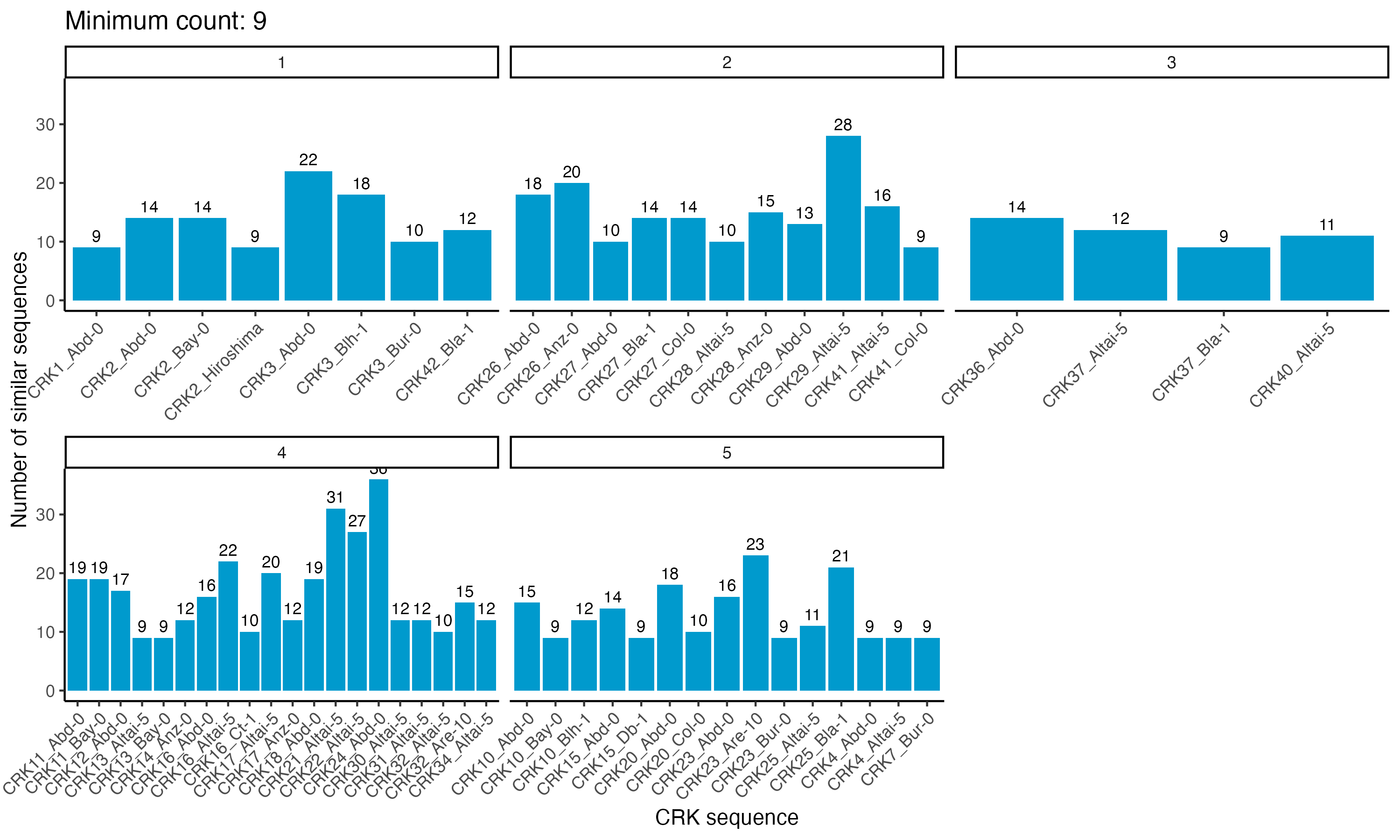

Supplement: Supplementary 1 — Figs. S1 to S8 Tables S1 and S2 Data S1 to S3 [file csbj.0043.f1.zip › SupplementaryFigure2.png]

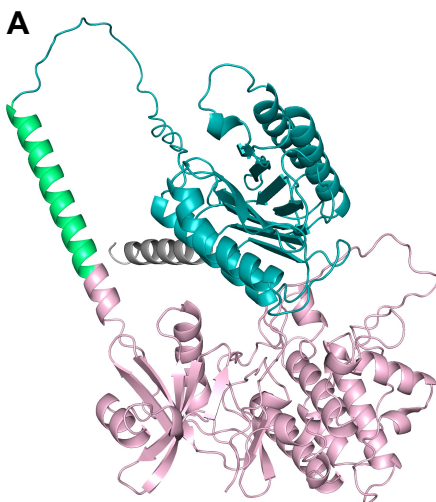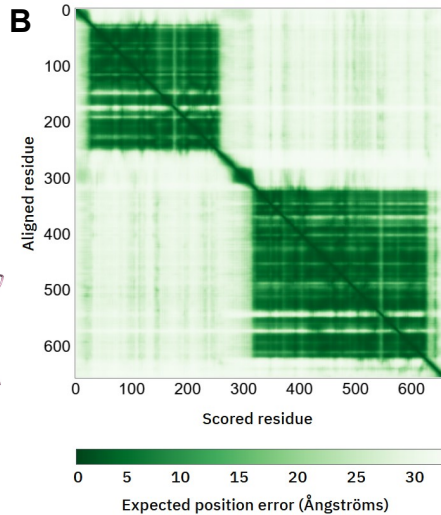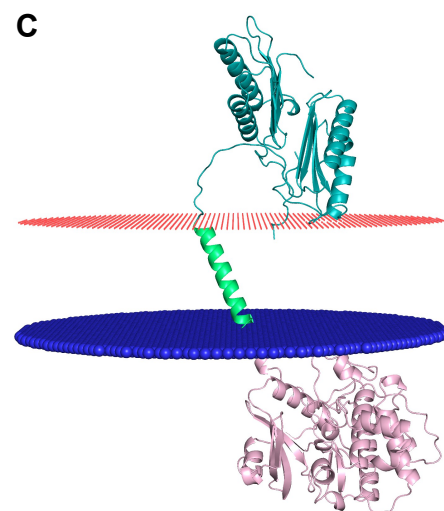

**D** Group I – CRK42

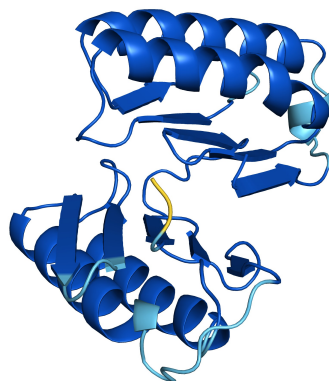

Group II – CRK29

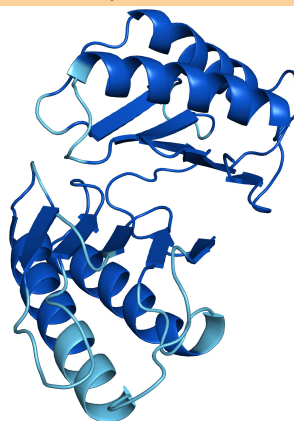

Group III – CRK39

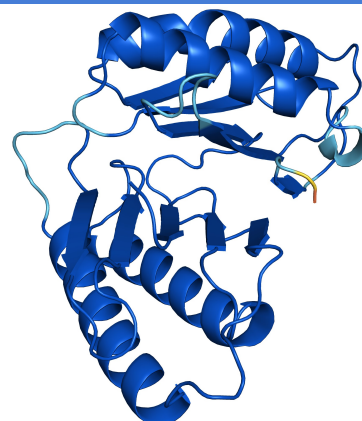

Group IV – CRK11

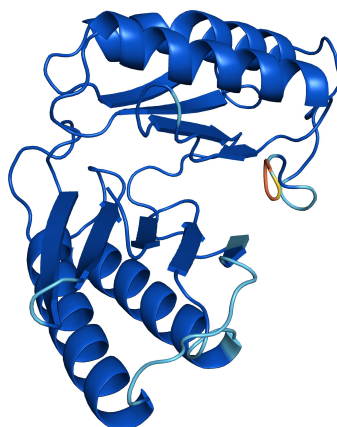

Group V – CRK8

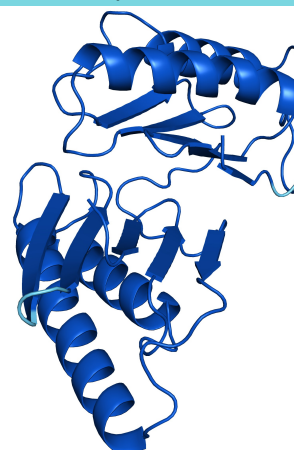

pLDDT Confidence Score:

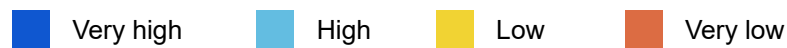

Supplement: Supplementary 1 — Figs. S1 to S8 Tables S1 and S2 Data S1 to S3 [file csbj.0043.f1.zip › SupplementaryFigure5.pdf]

# Group I – CRK42

# Group II

## DUF26-A

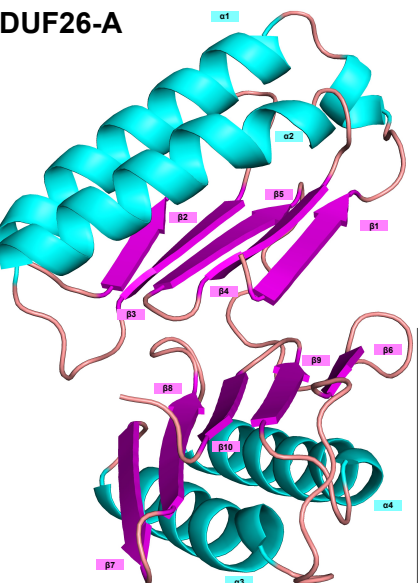

## DUF26-B

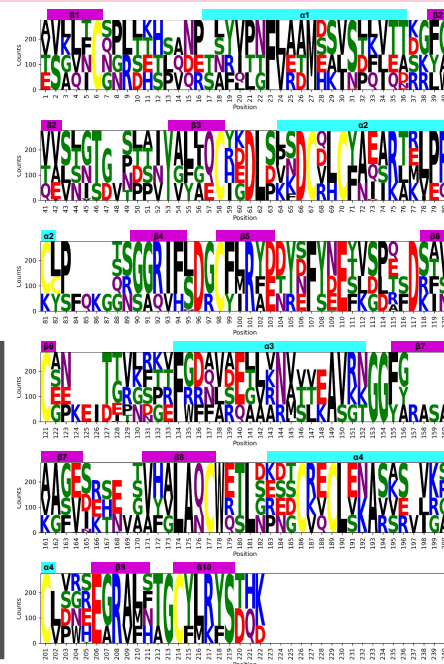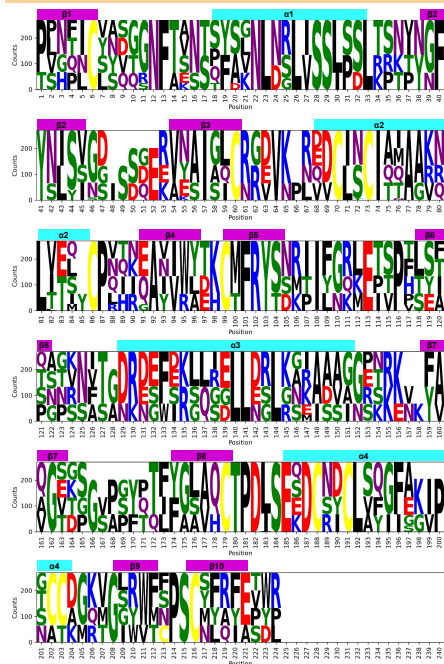

## Group III

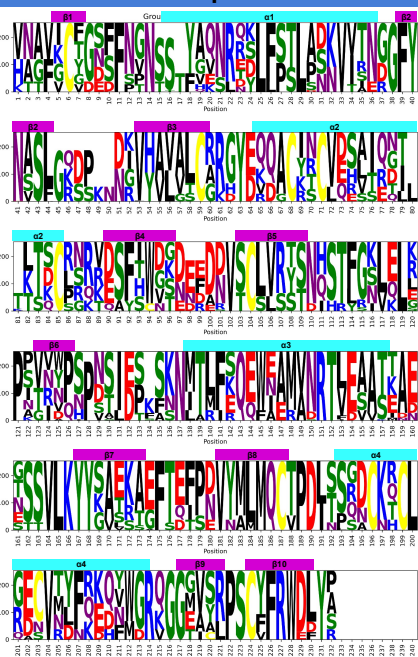

## Group IV

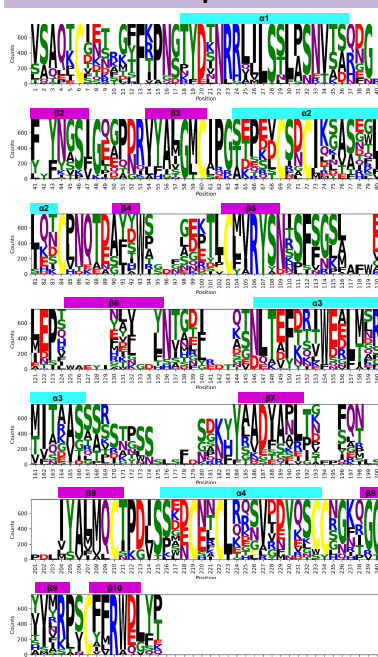

## Group V

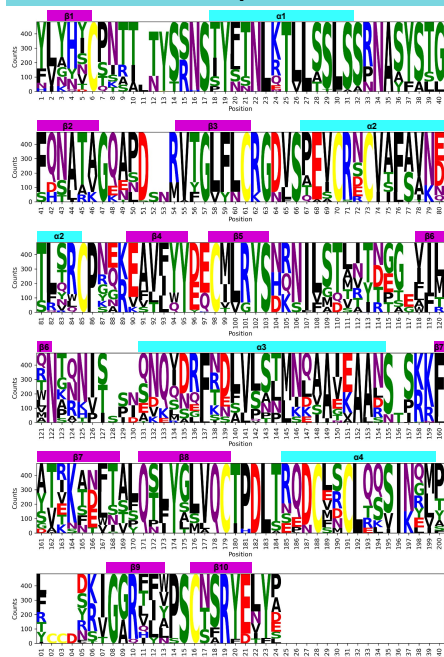

Supplement: Supplementary 1 — Figs. S1 to S8 Tables S1 and S2 Data S1 to S3 [file csbj.0043.f1.zip › SupplementaryFigure7.pdf]

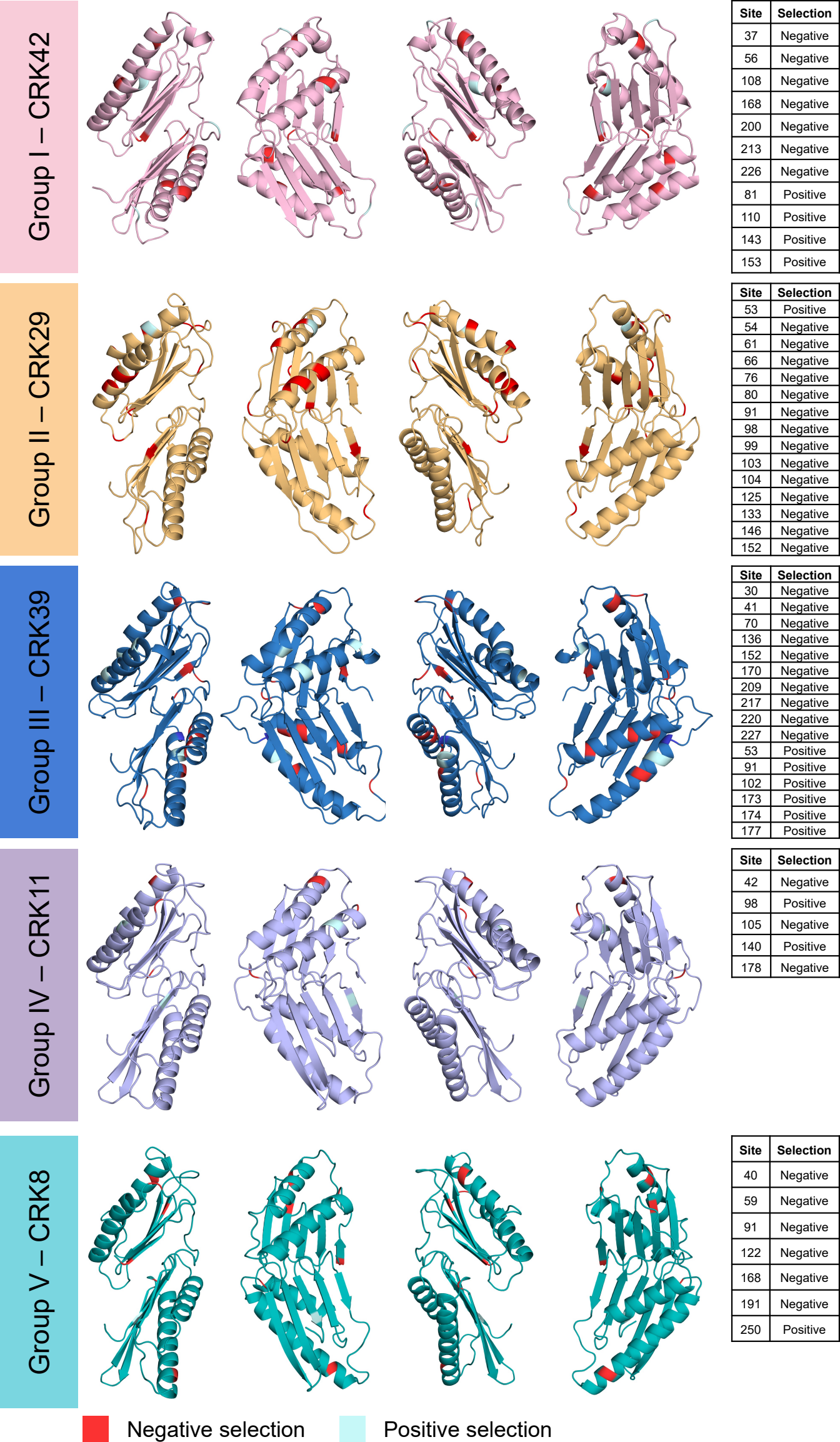

Supplement: Supplementary 1 — Figs. S1 to S8 Tables S1 and S2 Data S1 to S3 [file csbj.0043.f1.zip › SupplementaryFigure8.pdf]
