# Supplementary material for: Genetic Diversity, Predictive Protein Structures, and Interaction Networks of Cysteine-Rich Receptor-Like Kinases in Arabidopsis thaliana
Source: Comput Struct Biotechnol J. 2026 Apr 8;35(1):0043. doi: 10.34133/csbj.0043 (PMC13058244; doi:10.34133/csbj.0043)
Supplement: Supplementary 1 — Figs. S1 to S8 Tables S1 and S2 Data S1 to S3 [file csbj.0043.f1.zip › Supplementary data_sequences.zip!/Supplementary data_sequences/Supplementary Table 1.docx]

**Supplementary1** - List of CRK identifiers

| CRK | uniprot_ID | TAIR | TAIR_representative | gene_length | strand | synteny |
| --- | --- | --- | --- | --- | --- | --- |
| CRK1 | Q9LMB9 | AT1G19090 | AT1G19090.1 | 2266 | positive | solo |
| CRK2 | Q9CAL3 | AT1G70520 | AT1G70520.1 | 2447 | negative | clusterG |
| CRK3 | Q9CAL2 | AT1G70530 | AT1G70530.1 | 2630 | negative | clusterG |
| CRK4 | Q9LZU4 | AT3G45860 | AT3G45860.1 | 2641 | negative | solo |
| CRK5 | Q9C5S8 | AT4G23130 | At4g23130.2 | 2447 | negative | solo |
| CRK6 | Q9C5S9 | AT4G23140 | At4g23140.2 | 2641 | positive | solo |
| CRK7 | Q8L7G3 | AT4G23150 | AT4G23150.1 | 2571 | positive | solo |
| CRK8 | O65468 | AT4G23160 | AT4G23160.2 | 2762 | positive | solo |
| CRK10 | Q8GYA4 | AT4G23180 | AT4G23180.1 | 2610 | positive | clusterA |
| CRK11 | Q9ZP16 | AT4G23190 | AT4G23190.1 | 2514 | negative | clusterA |
| CRK12 | O65472 | AT4G23200 | AT4G23200.1 | 2555 | negative | clusterB |
| CRK13 | Q0PW40 | AT4G23210 | AT4G23210.3 | 2527 | negative | clusterB |
| CRK14 | Q8H199 | AT4G23220 | AT4G23220.1 | 2791 | negative | clusterC |
| CRK15 | Q8W4G6 | AT4G23230 | AT4G23230.1 | 2453 | negative | clusterC |
| CRK16 | O65476 | AT4G23240 | AT4G23240.1 | 2692 | negative | clusterC |
| CRK17 | Q8L710 | AT4G23250 | AT4G23250 | 2576 | negative | clusterC |
| CRK18 | Q8RX80 | AT4G23260 | AT4G23260.1 | 2528 | negative | clusterC |
| CRK19 | Q8GWJ7 | AT4G23270 | AT4G23270 | 2913 | negative | clusterC |
| CRK20 | O65479 | AT4G23280 | AT4G23280 | 2732 | positive | clusterC |
| CRK21 | Q3E9X6 | AT4G23290 | At4g23290.2 | 2901 | negative | clusterC |
| CRK22 | Q6NQ87 | AT4G23300 | AT4G23300.1 | 2530 | positive | clusterC |
| CRK23 | O65482 | AT4G23310 | AT4G23310.1 | 3027 | positive | clusterC |
| CRK24 | O65483 | AT4G23320 | AT4G23320 | 2109 | negative | clusterC |
| CRK25 | Q9M0X5 | AT4G05200 | AT4G05200 | 2517 | negative | solo |
| CRK26 | Q9T0J1 | AT4G38830 | AT4G38830.1 | 2605 | positive | solo |
| CRK27 | O49564 | AT4G21230 | AT4G21230.1 | 2436 | negative | solo |
| CRK28 | O65405 | AT4G21400 | AT4G21400.1 | 2492 | negative | clusterD |
| CRK29 | Q8S9L6 | AT4G21410 | AT4G21410 | 2188 | negative | clusterD |
| CRK30 | Q9LDT0 | AT4G11460 | AT4G11460.1 | 2626 | positive | clusterE |
| CRK31 | Q9LDM5 | AT4G11470 | AT4G11470 | 2433 | positive | clusterE |
| CRK32 | Q9LDS6 | AT4G11480 | AT4G11480.1 | 2392 | positive | clusterE |
| CRK33 | Q9LDN1 | AT4G11490 | AT4G11490.1 | 2701 | positive | clusterE |
| CRK34 | Q9LDQ3 | AT4G11530 | AT4G11530.1 | 2507 | positive | solo |
| CRK36 | Q9XEC6 | AT4G04490 | AT4G04490.1 | 2682 | negative | clusterF |
| CRK37 | Q9XEC7 | AT4G04500 | AT4G04500.1 | 2357 | positive | clusterF |
| CRK38 | Q9XEC8 | AT4G04510 | AT4G04510 | 2535 | positive | clusterF |
| CRK39 | Q9SYS7 | AT4G04540 | AT4G04540.1 | 2559 | positive | clusterF |
| CRK40 | Q9SYS3 | AT4G04570 | AT4G04570.1 | 2673 | positive | clusterF |
| CRK41 | O23081 | AT4G00970 | AT4G00970.1 | 3258 | positive | solo |
| CRK42 | Q9FNE1 | AT5G40380 | AT5G40380.1 | 2918 | positive | solo |
